# Thyroid Hormone Transporters MCT8 and OATP1C1 Are Expressed in Projection Neurons and Interneurons of Basal Ganglia and Motor Thalamus in the Adult Human and Macaque Brains

**DOI:** 10.3390/ijms24119643

**Published:** 2023-06-01

**Authors:** Ting Wang, Yu Wang, Ana Montero-Pedrazuela, Lucía Prensa, Ana Guadaño-Ferraz, Estrella Rausell

**Affiliations:** 1 School of Medicine, Department Anatomy Histology & Neuroscience, Autónoma de Madrid University (UAM), 28029 Madrid, Spain; ting.wang02@estudiante.uam.es (T.W.); yu.wang01@estudiante.uam.es (Y.W.); lucia.prensa@uam.es (L.P.); 2 PhD Program in Neuroscience, Autónoma de Madrid University (UAM)-Cajal Institute, 28029 Madrid, Spain; 3 Instituto de Investigaciones Biomédicas Alberto Sols, Consejo Superior de Investigaciones Científicas (CSIC)-Autónoma de Madrid University (UAM), 28029 Madrid, Spain; amontero@iib.uam.es

**Keywords:** thyroid hormones, thyroid hormone transporters, MCT8, OATP1C1, human, monkey, basal ganglia, motor thalamus, MSN cells, nucleus basalis of Meynert

## Abstract

Monocarboxylate transporter 8 (MCT8) and organic anion-transporting polypeptide 1C1 (OATP1C1) are thyroid hormone (TH) transmembrane transporters relevant for the availability of TH in neural cells, crucial for their proper development and function. Mutations in MCT8 or OATP1C1 result in severe disorders with dramatic movement disability related to alterations in basal ganglia motor circuits. Mapping the expression of MCT8/OATP1C1 in those circuits is necessary to explain their involvement in motor control. We studied the distribution of both transporters in the neuronal subpopulations that configure the direct and indirect basal ganglia motor circuits using immunohistochemistry and double/multiple labeling immunofluorescence for TH transporters and neuronal biomarkers. We found their expression in the medium-sized spiny neurons of the striatum (the receptor neurons of the corticostriatal pathway) and in various types of its local microcircuitry interneurons, including the cholinergic. We also demonstrate the presence of both transporters in projection neurons of intrinsic and output nuclei of the basal ganglia, motor thalamus and nucleus basalis of Meynert, suggesting an important role of MCT8/OATP1C1 for modulating the motor system. Our findings suggest that a lack of function of these transporters in the basal ganglia circuits would significantly impact motor system modulation, leading to clinically severe movement impairment.

## 1. Introduction

Thyroid hormones (TH) are essential for human brain development and functionality [1]. The active form, 3,5,3′-triiodothyronine (T3), modulates gene expression by directly binding to specific nuclear receptors, while indirect genomic actions mediated by T4 (3,5,3′,5′-tetraiodothyronine or thyroxine) or T3 by means of its binding to different receptors at the cytoplasmic membrane, cytoplasm, and mitochondria have also been described [2,3]. Experimental studies in rodents have established that TH availability and action in the central nervous system (CNS) are tightly controlled by two main mechanisms: local deiodination of TH, which activates and inactivates TH inside the brain [4,5], and TH transport across the cell membranes [6,7,8]. Several TH transporters have been described, and an earlier review by our group provided a detailed summary of these transporters, their functions, and their clinical implications [8]. Briefly, there are two widely accepted transporters with physiological functions in the CNS: the monocarboxylate transporter 8 (MCT8) [6] and the organic anion-transporting polypeptide 1C1 (OATP1C1) [7] belonging to the MCT and OATP families, respectively. Both are included in the major facilitator superfamily (MFS), with elements that show a double bundle of six transmembrane domains joined with a large intracellular loop. MCT8 has a high affinity for T4 and its nuclear active form T3 [6], while OATP1C1 transports T4, with the highest affinity among all known TH transporters [8], as well as the iodothyronine product of its deiodination, reverse T3 (rT3) [7]. OATP1C1 also transports other substances such as metabolites of steroid hormones, including 17-(β-D-glucuronic acid) estradiol, but virtually no T3 [7].

Mutations in the MCT8 gene (*SLC16A2*) and in the OATP1C1 gene (*SLCO1C1*) can cause a psychomotor developmental delay in humans. MCT8 deficient patients show a specific TH peripheral blood profile with high T3, low T4, low rT3, and normal to slightly elevated thyrotropin (TSH), whereas OATP1C1-deficient patients present hypothyroid symptoms such as intolerance to cold but with a normal thyroid function test in serum. MCT8 mutations located on the X chromosome are linked to the rare disease known as MCT8 deficiency or Allan–Herndon–Dudley Syndrome (AHDS) [9,10]. Aside from severe cognitive impairment, AHDS patients present noticeable neuromotor disturbances, such as central hypotonia, pyramidal signs such as Babinski (referable to failure in cortical commands), and extrapyramidal signs such as dystonia, choreoathetosis, and hypokinesia (referable to failure of the basal ganglia inhibitory–excitatory commands) [11,12,13]. General delay in myelination or myelin dysplasia has been shown in AHDS patients by magnetic resonance imaging (MRI) [10,11,14,15,16,17,18] and histopathological examinations. The first and sole patient with a functional mutation in OATP1C1 was reported in 2018 [19] in a 15.5-year-old girl showing gradual deterioration of cognitive and motor domains, with gait apraxia, myocloniclike movements in the hands, scoliosis, and spasticity of the lower limbs. Brain imaging with MRI and positron emission tomography–computed tomography demonstrated grey and white matter degeneration and severe glucose hypometabolism [19].

Patients with TH transporter deficiency suffer from severe motor disturbances that can be attributed to dysfunction of the cerebellum and its related nuclei, dysfunctions of the cortical command, and dysfunctions of the basal ganglia. The study of the expression of TH transporters in those systems is relevant to understanding the physiopathogenesis of the disease. In previous work, we have discussed the alterations of the cerebellum in AHDS patients [20] as a possible underlying factor for motor disturbances. We have also discussed the possible implications of the lack of expression of MCT8 and OATP1C1 in cortical projection neurons and interneurons of adult human and monkey brains [21]. Here we focus on the distribution of TH transporters in the basal ganglia.

**Figure 1:**
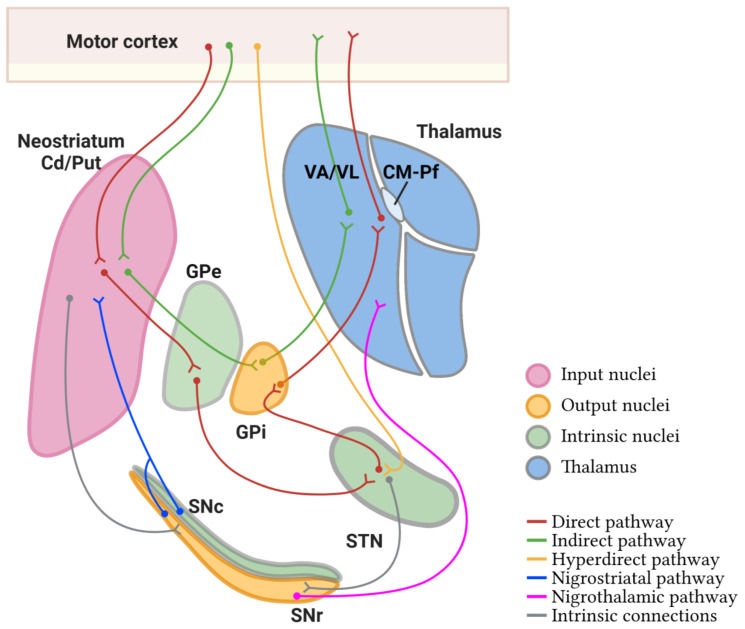
Diagram of the definition of basal ganglia nuclei according to their connectivity. The motor circuits are illustrated in different colors. Cd/Put: caudate nucleus and putamen, CM-Pf: centromedian–parafascicular nuclei of the thalamus, GPe: external segment of globus pallidus, GPi: internal segment of globus pallidus, SNc: substantia nigra pars compacta, SNr: substantia nigra pars reticulata, STN: subthalamic nucleus, VA/VL: ventral anterior and ventral lateral nuclei of the thalamus. (Created with BioRender.com).

Basal ganglia (basal nuclei) are a group of interconnected subcortical nuclei that are primarily responsible for the modulation of motor control [22]. The definition of the cell groups comprising the basal ganglia has been revised over the years with contemporary views focusing on the functional characteristics of these nuclei. In this respect, the basal ganglia consist of (1) the caudate, putamen, and globus pallidus (together forming the dorsal basal nuclei), (2) the nucleus accumbens plus part of the adjacent olfactory tubercle (the ventral striatum), and (3) the substantia innominata or nucleus basalis of Meynert (ventral pallidum). Another way of grouping these nuclei is based on their connectional circuits with the motor system. Figure 1 shows a simplification of this concept [23]: (1) input nuclei, which refers to the dorsal striatum (consisting of the caudate nucleus (Cd) and the putamen (Put)) and the ventral striatum (accumbens nucleus, not covered in this article); (2) output nuclei, consisting of the internal segment of the globus pallidus (GPi) and the substantia nigra pars reticulata (SNr); and (3) intrinsic nuclei, consisting of the external segment of the globus pallidus (GPe), the substantia nigra pars compacta (SNc), and the subthalamic nucleus (STN). Input nuclei receive information from various sources, primarily the motor cortex, the centromedian–parafascicular nucleus (CM-Pf) of the thalamus, and the substantia nigra; intrinsic nuclei relay information from input nuclei to output nuclei; and output nuclei send basal ganglia-processed information to the motor thalamus, mainly the ventral anterior and ventral lateral nuclei (VA/VL), which in turn are connected to the motor cortex. The classical basal ganglia model depicts how the commands that lead to a coordinated sequence of muscle contractions produce a movement flow from the motor cortex through the basal ganglia and back to the cortex.

Briefly, the corticospinal motor command for muscle contraction is relayed by collaterals of the pyramidal pathway to the striatum in the basal ganglia. These nuclei contain the representation of the whole final movement scenario (because they receive connections from all the association cortical areas) and generate new orders that need to go through several synapses in the intrinsic and output nuclei and in the thalamus to prepare the cortex for the next sequence of motor commands, either inhibiting the previous command or exciting the output of the new command. The motor thalamus is a key intermediate element in this mechanism; it is an excitatory pathway for the motor cortex (and movement) unless it is inhibited by the basal ganglia, similarly to the way a jockey controls a horse with the reins. This allows the preparation of the cortex for the sequential and fast release of cortical motor commands one after the other, and thence, a coordinated sequence of muscle contractions for movement. Disruptions in the basal ganglia network cause several movement disorders, mainly including: (1) hypokinetic movements (akinesia and bradykinesia), (2) hyperkinetic movements (ballism, chorea, and athetosis), and (3) dystonia (characterized by prolonged muscle spasms and abnormal postures) [24,25].

The cortex delivers the information to the striatum (caudate nucleus and putamen which have the same cytoarchitecture) via the corticostriatal pathway. This mainly ends in one kind of neuron, the medium-sized spiny neurons (MSN), which are GABAergic projection neurons, and in other interneurons that in turn modulate MSN [23]. In all striatal subdivisions, the inhibitory projection of MSN is the origin of the two main circuits to the thalamus [26] as illustrated in Figure 1: (a) the direct pathway formed by the inhibitory axons of MSN to the GPi and SNr (two output nuclei), whose projections to the thalamus are inhibitory, resulting in a disinhibition of the motor thalamus and facilitation of the thalamocortical action for movement, and (b) the indirect pathway formed by the inhibitory axons of MSN to the GPe (an intrinsic nucleus), which in turn sends inhibitory axons to another intrinsic nucleus, the STN, resulting in disinhibition of the STN. The STN in turn projects an excitatory connection back to the GPi and SNr, which in turn activate inhibitory projections to the motor thalamus, thus resulting in a deactivation of the thalamocortical pathway and a disfacilitation of the motor command. MSN that originate one or the other pathway are distinguished by their expression of dopamine receptors. MSN that express dopamine receptor type 1 (DRD1) are called D1 receptor-expressing MSN (D1-MSN) and start the direct pathway, while MSN that express dopamine receptor type 2 (DRD2) are called D2 receptor-expressing MSN (D2-MSN) and start the indirect pathway [23,27]. Additionally, there is a local striatal microcircuitry formed by cholinergic and several types of GABAergic interneurons that influence MSN activity both directly and indirectly through influences on corticostriatal, thalamostriatal, and nigral dopaminergic afferents [28]. Some groups of neostriatal GABAergic interneurons can be identified by their expression of calcium-binding proteins such as Calbindin-D-28K (CALB), Calretinin (CALR), or Parvalbumin (PARV) [29] and of neuropeptides such as Somatostatin (SOM) [30] and nitric oxide [30,31].

All the projection neurons in the intrinsic and output nuclei of the basal ganglia–thalamic circuits are GABAergic [32,33,34] except those in the SNc, which are dopaminergic, [34] and in the STN [35] and the VA/VL of the thalamus, which are glutamatergic [36]. All of them can also be identified by their expression of PARV in GPe, GPi, STN, and SNr [32,33,34,35] and *core* thalamocortical cells of VA/VL or CALB in *matrix* thalamocortical cells of the VA/VL [37,38] or dopamine in SNc [34].

Syndromes caused by deficiency of the TH transporters MCT8 and OATP1C1 [11,12,19] evolve with clinical disorders that point to lesions in the basal ganglia and disruptions in both direct and indirect circuitries. It is then reasonable to hypothesize that the neurons involved in basal ganglia circuits might express these transporters and that their absence causes alterations in the balance of excitation/inhibition in the striatum microcircuitry and in the activity balance between the neurons of the direct and indirect circuits, contributing to the clinical picture described. However, there is no previous information regarding the precise cellular location of MCT8 and OATP1C1 in these circuits. Previous in situ hybridization studies have demonstrated mRNA expression of Mct8 in the adult mouse basal ganglia [39,40], but there is no reference to specific cell types. Human multiple tissue northern blots [7] have shown high expression of OATP1C1 mRNA in the striatum and moderate expression in other nuclei of the basal ganglia and thalamus. The large-scale human study Genotype–Tissue Expression (GTEx) project [41] shows that the median value of the MCT8 gene *SLC16A2* transcripts per million (TPM) is moderately high in basal ganglia among other brain structures while the TPM values of the OATP1C1 gene *SLCO1C1* are extremely high in comparison with other tissues. Analyses of human brain transcriptome data sets show high MCT8 expression in the caudate nucleus and nucleus basalis of Meynert [42]. To date, the study of the protein distribution of these TH transporters in adult human basal ganglia and thalamus has been very unspecific, with a lack of information defining the precise filiation of the transporter-expressing neurons.

Even though further procedures for TH transporters deficiency syndromes are being developed [43], effective treatments for the neurological alterations remain to be investigated. It has become critical to determine the anatomical distribution of MCT8 and OATP1C1, as well as the exact nature of the cells and elements that express them, to model the etiopathogenesis of transporter-deficient syndromes and to advance in the development of therapeutic strategies.

In the present study, we analyzed the distribution of MCT8 and OATP1C1 in the projection neurons and interneurons in input, intrinsic, and output nuclei of the basal ganglia of adult humans and monkeys and in their blood–brain barriers, aiming at further exploring the role of MCT8- and OATP1C1-expressing cells in this important system of motor modulation and control.

We found that TH transporters MCT8 and OATP1C1 are widely expressed in all projection neurons of basal ganglia and related nuclei that participate in direct and indirect motor pathways, suggesting that a lack of function of these transporters in the basal ganglia circuits would have a significant impact on the motor system modulation, leading to clinically severe movement impairment.

## 2. Results

We analyzed the cellular expression of the TH transporters MCT8 and OATP1C1 in the nuclei of the basal ganglia in the brains of adult humans and monkeys, as specified in Table 1 and Table 2. We performed immunohistochemistry (IHC) and double/triple staining immunofluorescence (IF) following optimized protocols as described in the Methods section and used previously in Wang et al., 2023 [21]. We used a total of 100 coronal sections from seven adult human individuals for detecting MCT8 and 99 sections for detecting OATP1C1. These were taken every 2–4 mm in the antero-posterior extent of the striatum, to ensure that the GP, thalamus, STN, and other related nuclei were included in the section. In parallel, we used 87 sections of 10 monkeys to detect MCT8 and 81 for OATP1C1. The sections were taken every 2–3 mm to include the basal ganglia in the sections. The above data include only the number of sections that were successfully examined. There are many more tissue sections used for experimental optimization, antibody testing, blank controls, etc. The brain tissue of one Squirrel monkey (*Saimiri sciureus*) was histologically analyzed. As we only analyzed one monkey from this genus and the results obtained were the same in terms of types of neurons and cells expressing MCT8 and OATP1C1 in all monkeys independently of the genus, only data from macaques are shown in the figures and throughout the text. All figures in this section and the Supplementary Materials section show the most representative results.

### 2.1. General Pattern of MCT8 and OATP1C1 Immunostaining in the Adult Human and Macaque Basal Ganglia Nuclei

**Figure 2:**
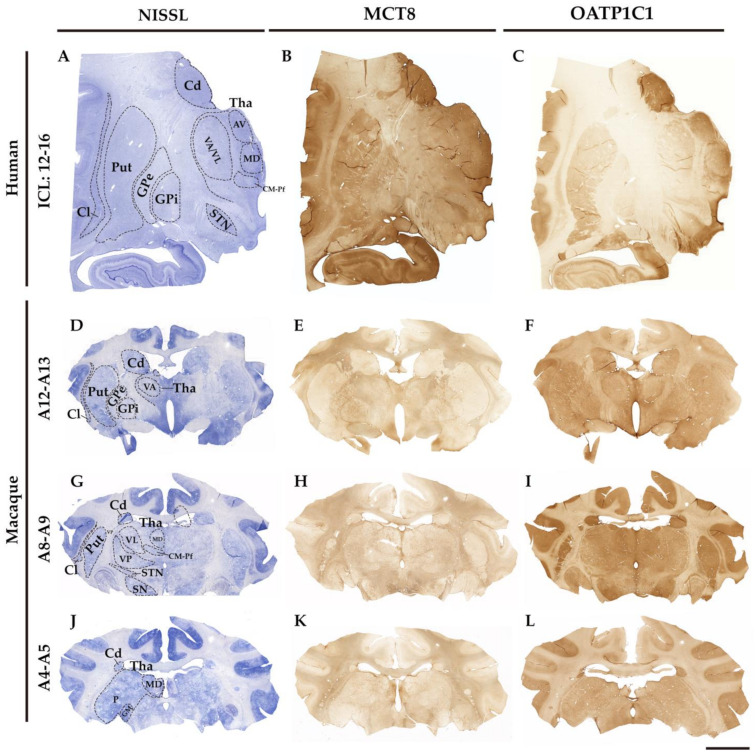
MCT8 and OATP1C1 expression profiles in human and macaque coronal sections of the thalamus and basal ganglia. Compositions show representative brightfield photomicrographs taken from Nissl-stained adjacent sections (left) to MCT8 (middle) and OATP1C1 (right) immunostaining in humans (**A**–**C**) and macaques (**D**–**L**), showing that both markers identify the architecture and outlines of thalamic and basal ganglia nuclei. The section level is indicated at the left side according to the atlases Mai, J. K., et al., 2015 (human) [44] and Winters, W. D., et al., 1969 (macaque) [45]. ICL, Inter-Commissural Line, (**A**–**C**) refers to the distance from the center of the anterior commissure. AV: Anterior ventral nucleus, Cd: caudate nucleus, Cl: claustrum, CM-Pf: Centro median–parafascicular nucleus, GM: Corpus geniculatum mediale, GPe: external segment of globus pallidus, GPi: internal segment of globus pallidus, MD: Dorso Median nucleus, P: Pulvinar nucleus, Put: putamen, SN: substantia nigra, STN: subthalamic nucleus, Tha: thalamus, VA: Ventral anterior nucleus, VL: Ventral lateral nucleus, VP: Ventral posterior nucleus. Scale bar = 8000 μm (**A**–**C**) and 9000 μm (**D**–**L**).

Figure 2 shows the general regional MCT8 and OATP1C1 immunohistochemical labeling patterns in coronal sections of the human and macaque monkey thalamus and basal ganglia nuclei. The negative controls are shown in Figure S1. MCT8 and OATP1C1 are differentially expressed across the brain regions.

At low magnification, the MCT8 immunostaining signal in the human section is seen in the caudate nucleus, putamen, claustrum, globus pallidus, STN, and thalamus (Figure 2B). The white matter also shows light positive signals, which reflect the labeling of fibers that are seen at higher magnification. In the macaque brain sections (Figure 2E,H,K), the MCT8 immunostaining signal is also present in the caudate–putamen complex, in the GPe and GPi, in the VA/VL, in the STN, and the substantia nigra, with a very clean signal that is better appreciated at higher magnification. White matter shows an intensely positive signal reflecting a very high number of thick fibers that are in general very intensely labeled and can be observed at higher magnification. The OATP1C1 immunostaining signal is observed in the same mentioned nuclei in human (Figure 2C) and macaque sections (Figure 2F,I,L).

**Figure 3:**
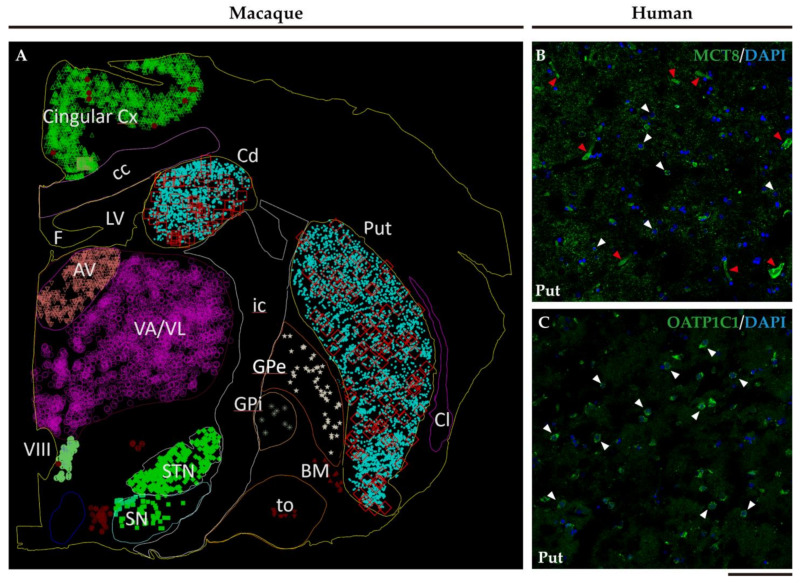
The topographic distribution of MCT8 and OATP1C1 at the human putamen. (**A**) Neurolucida plot from one of our monkey sections, showing the general distribution of MCT8 labeled cells. Note that labeled cells are sparsely distributed across all the basal ganglia nuclei and thalamus. (**B**) Composition of 2 × 2 confocal images from the human putamen at 60× each, showing a surface of 400 µm × 400 µm, taken from one MCT8 immunofluorescence-stained section. White arrowheads point to labeled cells. Red arrowheads point to vessels. (**C**) Composition of 2 × 2 confocal images from the human putamen at 60× each, showing a surface of 400 µm × 400 µm, taken from one OATP1C1 immunofluorescence-stained section. AV: Anterior ventral nucleus of thalamus, BM: Nucleus basalis of Meynert, cc: Corpus callosum, Cd: caudate nucleus, Cl: claustrum, Cx: cortex, F: Fornix, GL: Corpus geniculatum laterale, GPe: external segment of globus pallidus, GPi: internal segment of globus pallidus, ic: Internal capsule, LV: Lateral ventricle Put: putamen, SN: substantia nigra, STN: subthalamic nucleus, to: Tractus opticus, VA/VL: ventral anterior and ventral lateral nuclei of the thalamus, VIII: Third Ventricle, Bar = 2950 μm (**A**), 100 μm (**B**,**C**).

Figure 3A shows an example of a Neurolucida-generated plot with a representation of the general distribution of all labeled neurons in the basal ganglia nuclei and thalamus of an MCT8 DAB immunohistochemistry monkey section, created after a complete scanning at 20×. Note that the cells are sparsely distributed across the whole surface of the nuclei and do not form groups or clusters that could be related to the matrix/striosome structures (26). Figure 3B,C shows the distribution of the cells at 60×, in compositions generated with 2 × 2 confocal images to show a surface of 400 µm × 400 µm. Large and small positive cells can be observed sparsely distributed in both cases, but positive vessels are only observed in the MCT8 section.

### 2.2. MCT8 and OATP1C1 in Blood Vessels and Barriers in Macaque and Human Basal Ganglia

**Figure 4:**
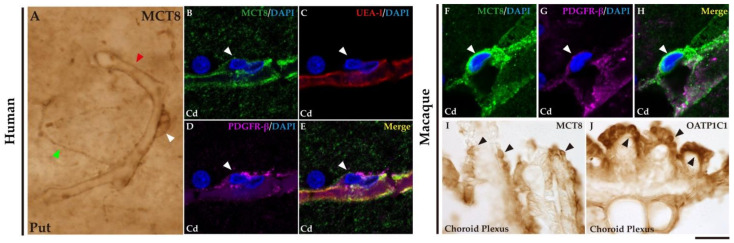
Expression of MCT8 and OATP1C1 in blood vessels and brain barriers in the human and macaque basal ganglia and adjacent choroid plexus. (**A**) Representative brightfield photomicrograph shows immunostaining for MCT8 in the human putamen. Note that an MCT8 immunopositive signal is observed along the capillary wall (red arrowhead), fibers (green arrowhead), and “bump-on-a-log” morphology pericytelike cells (white arrowhead). (**B**–**H**) Representative confocal microscope compositions from multiple-stained sections for MCT8 (green), the endothelial marker UEA-I (red), and the vascular and pericyte biomarker PDGFR-β (purple) in human and macaque caudate nucleus. Merged image (**E**,**H**) shows the colocalization of all signals. (**B**–**E**) Coexpression of MCT8, UEA-I, and PDGFR-β is observed in a vessel, while coexpression of MCT8 and PDGFR-β but not UEA-I is observed in a capillary-associated pericyte (white arrowheads) in humans. (**F**–**H**) Coexpression of MCT8 and PDGFR-β in a vessel and pericytelike cells (white arrowheads) in macaques. Counterstaining with DAPI (blue) shows nuclei of all cells. (**I**,**J**) Representative brightfield photomicrographs show immunostaining for MCT8 (**I**) and OATP1C1 (**J**) in the macaque choroid plexus at the lateral ventricle. Black arrowheads point to ependymocytes. Cd: caudate nucleus, Put: putamen, PDGFR-β: platelet-derived growth factor receptor-β, UEA-I: *Ulex europaeus* agglutinin-I. Scale bar = 10 μm (**A**–**H**) and 50 μm (**I**,**J**).

We investigated the expression of MCT8 and/or OATP1C1 in the blood vessels and barriers in the adult human and macaque basal ganglia. Consistent with previous studies [46,47], we found that MCT8 immunostaining is widely distributed in the blood vessels of all the studied nuclei (Figure 4A), whereas OATP1C1 immunostaining is very rare. Figure 4B–E shows in humans the colocalization of MCT8, *Ulex europaeus* agglutinin-I (UEA-I) lectin that labels endothelial cells, and platelet-derived growth factor receptor-β (PDGFR-β) that was used to label all vascular mural cells. Figure 4B–E shows the colocalization of MCT8 in perivascular cell profiles with PDGFR-β but not with UEA-I, indicating that those MCT8 immunopositive profiles are pericytes. Those profiles can also be observed in the macaque (Figure 4F–H). In addition, the expression of MCT8 and OATP1C1 was observed in the choroid plexus of the ventricles in the macaque brain (Figure 4I,J).

### 2.3. MCT8 and OATP1C1 Are Expressed in Input Nuclei of the Human and Macaque Basal Ganglia

**Figure 5:**
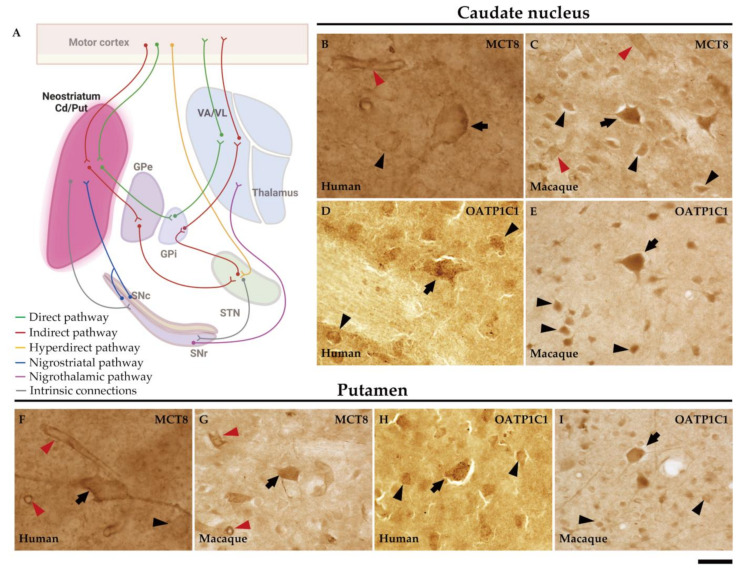
Expression of MCT8 and OATP1C1 in neurons of the human and macaque striatum. (**A**) The diagram summarizes the basal ganglia motor circuits. The striatum (caudate nucleus and putamen) serves as the input nuclei of the basal ganglia, receiving the information primarily from the motor cortex. (**B**–**I**) Representative brightfield photomicrographs show immunostaining for MCT8 in the human (**B**) and macaque (**C**) caudate nucleus and in the human (**F**) and macaque (**G**) putamen. OATP1C1 immunostaining is also shown in human (**D**) and macaque (**E**) caudate nucleus and the human (**H**) and macaque (**I**) putamen. Note that MCT8 and OATP1C1 are observed in neurons of different sizes and that the MCT8 signal, but not the OATP1C1 signal, is observed in capillaries (red arrowheads). Black arrows point to large-sized neurons with immunopositive signals in the soma and dendrites. Black arrowheads point to medium/small-sized immunopositive neural cells. Cd/Put: caudate nucleus and putamen, GPe: external segment of globus pallidus, GPi: internal segment of globus pallidus, SNc: substantia nigra pars compacta, SNr: substantia nigra pars reticulata, STN: subthalamic nucleus, VA/VL: ventral anterior and ventral lateral nuclei of the thalamus. ((**A**) created with BioRender.com). Scale bar = 25 μm (**B**), 33 μm (**C**–**E**), 29 μm (**F**) and 38 μm (**G**–**I**).

The striatum (caudate nucleus and putamen) are the input nuclei of the motor pathway of the basal ganglia in humans and macaques (Figure 5A). In the human striatum, MCT8 immunoreactivity is present at the soma, cell membrane and dendrites of medium-sized neurons and in the soma of the large-sized neurons, as well as diffusely distributed in the extracellular matrix or neuropil (Figure 5B,F). Likewise, the neuronal expression of MCT8 is also observed in the macaque striatum at the cell membrane, cytoplasm, and dendrites (Figure 5C,G) of medium and large cells. The OATP1C1-immunopositive signal is high in medium-sized and large neurons in humans (Figure 5D,H) and macaques (Figure 5E,I), which show intense signals in the soma and dendrites. By their morphology, we can identify these medium-sized MCT8 and OATP1C1-immunopositive neurons either as MSN (projection neurons) or as interneurons, while the large-sized cells might correspond to cholinergic neurons.

**Figure 6:**
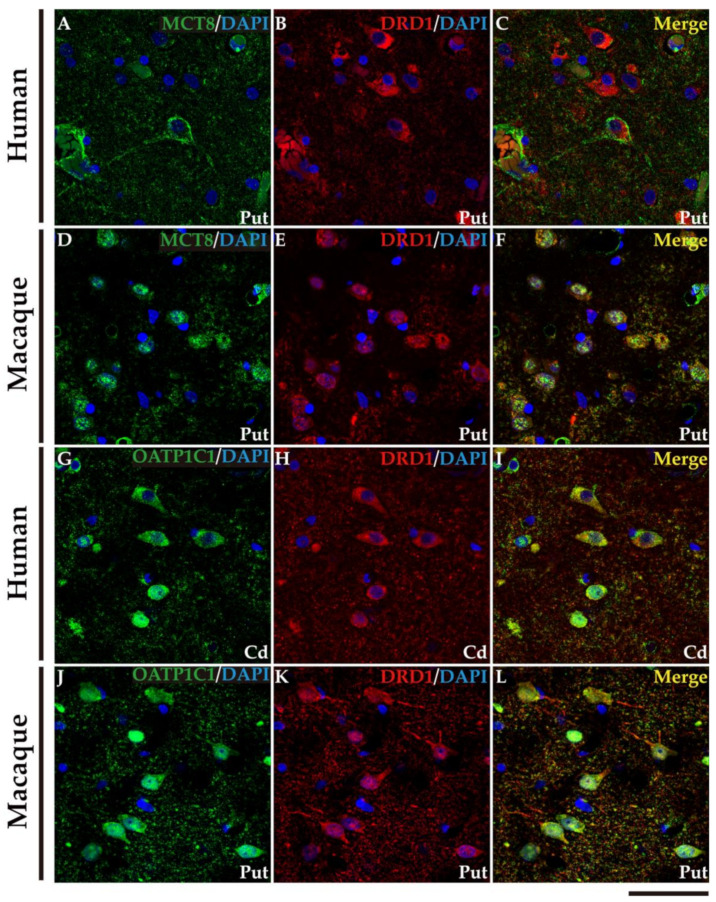
Expression of MCT8 and OATP1C1 in D1-MSN (direct pathway medium-sized spiny neurons) in human and macaque striatum. Representative confocal microscope compositions from double-stained sections for MCT8 (green) or OATP1C1 (green) and for the D1-MSN marker DRD1 (red) in caudate nucleus or putamen. Merged images (right side) show the colocalization of both signals. Coexpression of MCT8 and DRD1 is observed in human (**A**–**C**) and macaque (**D**–**F**) striatum. Coexpression of OATP1C1 and DRD1 is observed in human (**G**–**I**) and macaque (**J**–**L**) striatum. Counterstaining with DAPI (blue) shows nuclei of all cells. Note that in humans the MCT8 signal is located mainly at the cell membrane, while in macaques it is located at the membrane and in the cytoplasm. Cd: caudate nucleus; DRD1: Dopamine receptor type 1; D1-MSN: D1 receptor-expressing medium-sized spiny neurons; Put: putamen. Scale bar = 50 μm.

**Figure 7:**
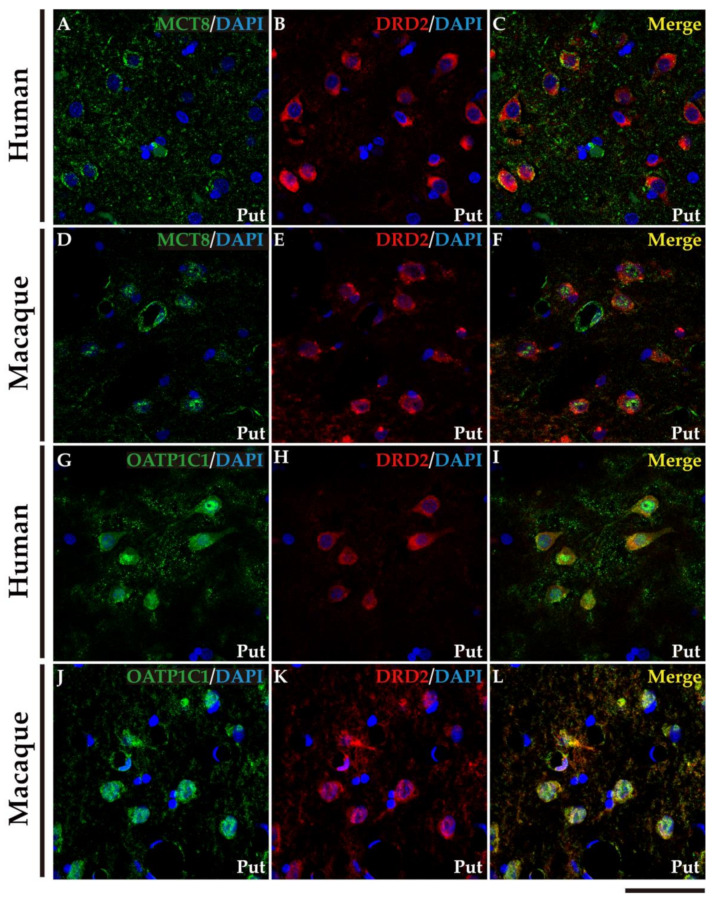
Expression of MCT8 and OATP1C1 in D2-MSN (indirect pathway medium-sized spiny neurons) in human and macaque striatum. Representative confocal microscope compositions from double-stained sections for MCT8 (green) or OATP1C1 (green) and for the D2-MSN marker DRD2 (red) in the putamen. Merged images (right side) show the colocalization of the two signals. Coexpression of MCT8 and DRD2 is observed in human (**A**–**C**) and macaque (**D**–**F**) striatum. Coexpression of OATP1C1 and DRD2 is observed in human (**G**–**I**) and macaque (**J**–**L**) striatum. Counterstaining with DAPI (blue) shows nuclei of all cells. Note that in humans, the MCT8 signals are located mainly at the cell membrane, while in macaques, they are also located in the cytoplasm. D2-MSN: D2 receptor-expressing medium-sized spiny neurons; DRD2: Dopamine receptor type 2; Put: putamen. Scale bar = 50 μm.

We performed double labeling IF studies in human and macaque sections to further address the nature of those cells. Our analyses show that MCT8 and OATP1C1 immunostaining colocalize in the medium-sized cells with typical MSN biomarkers (Figure 6 and Figure 7). MCT8 and OATP1C1 are coexpressed with the DRD1 in human and macaque caudate and putamen (Figure 6) and also with DRD2 (Figure 7). This indicates that MCT8 and OATP1C1 are expressed in MSN cells in both the direct pathway (D1-MSN, Figure 6), and the indirect pathway (D2-MSN, Figure 7). In humans, the cellular MCT8 signal is mainly restricted to the membrane and dendrites consistently with our IHC results (Figure 5).

**Figure 8:**
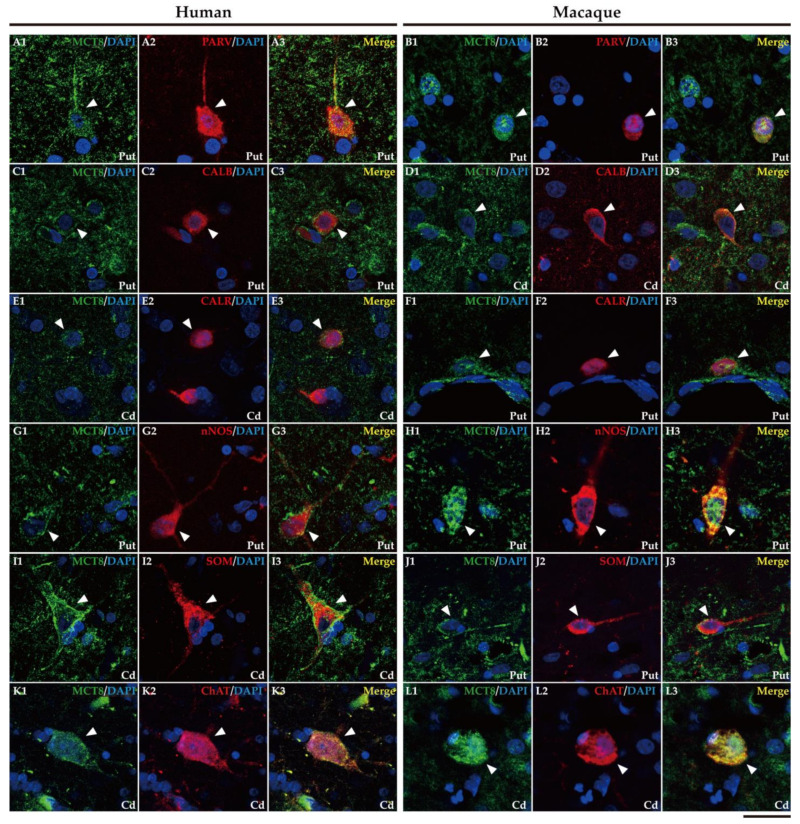
Expression of MCT8 in different subpopulations of neostriatal interneurons. Representative confocal microscope compositions of human (left) and macaque (right) double-stained sections for MCT8 and several markers to visualize different subtypes of interneurons. Merged images show the colocalization of both signals. (**A1**–**B3**) Coexpression of MCT8 (green) with the interneuron marker PARV (red) in fast-spiking GABAergic interneurons in the human and macaque putamen. (**C1**–**D3**) Coexpression of MCT8 (green) with the interneuron marker CALB (red) in human putamen and macaque caudate nucleus. Note that MCT8 expression was mainly seen at the cell membrane as well as in the neuropil. (**E1**–**F3**) Coexpression of MCT8 (green) with the interneuron marker CALR (red) in the human caudate nucleus and macaque putamen. (**G1**–**H3**) Coexpression of MCT8 (green) with nNOS (red) in nitrergic interneurons and persistent low-threshold spiking interneurons in the human and macaque putamen. (**I1**–**J3**) Coexpression of MCT8 (green) with SOM (red) in persistent low-threshold spiking interneurons in the human caudate nucleus and macaque putamen. (**K1**–**L3**) Coexpression of MCT8 (green) with ChAT (red) at the soma and processes of cholinergic interneurons (tonically active interneurons) in the human and macaque caudate nucleus. Note that the coexpression of all these markers with MCT8 is only partial. Counterstaining with DAPI (blue) shows nuclei of all cells. White arrowheads point to double-labeled interneurons. CALB: Calbindin-D-28K, CALR: Calretinin, Cd: caudate nucleus, ChAT: Choline Acetyltransferase, nNOS: Neuronal nitric oxide synthase, PARV: Parvalbumin, Put: putamen, SOM: Somatostatin. Scale bar = 25 μm.

**Figure 9:**
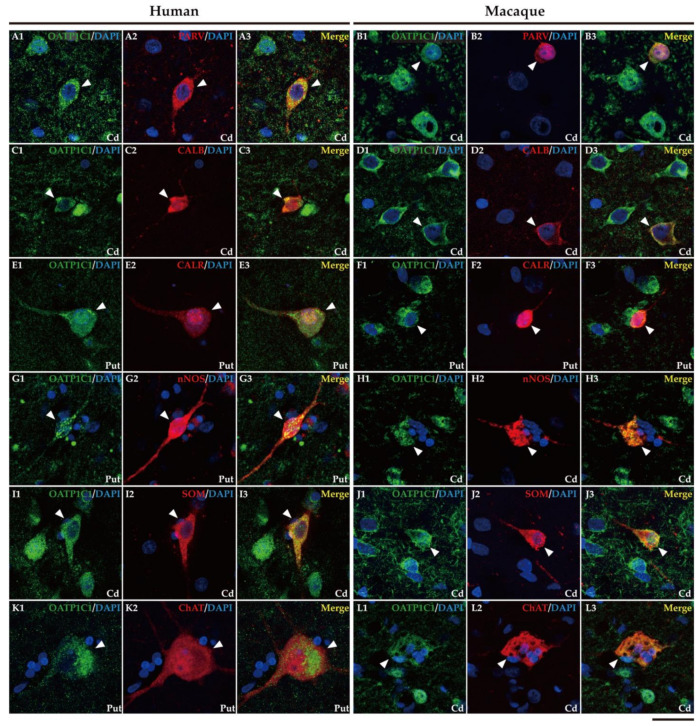
Expression of OATP1C1 in different subpopulations of interneurons in the striatum. Representative confocal microscope compositions of human (left) and macaque (right) double-stained sections for OATP1C1 and several markers to visualize different subsets of interneurons. Merged images show the colocalization of the two signals. (**A1**–**B3**) Coexpression of OATP1C1 (green) with the marker PARV (red) in fast-spiking GABAergic interneurons in the human and macaque caudate nucleus. (**C1**–**D3**) Coexpression of OATP1C1 (green) with the interneuron marker CALB (red) in the human and macaque caudate nucleus. (**E1**–**F3**) Coexpression of OATP1C1 (green) with the interneuron marker CALR (red) in human and macaque putamen. (**G1**–**H3**) Coexpression of OATP1C1 (green) with nNOS (red) in nitrergic interneurons and persistent low-threshold spiking interneurons in human putamen and macaque caudate nucleus. (**I1**–**J3**) Coexpression of OATP1C1 (green) with SOM (red) in persistent low-threshold spiking interneurons in the human and macaque caudate nucleus. (**K1**–**L3**) Coexpression of OATP1C1 (green) with ChAT (red) at the soma and processes of cholinergic interneurons (tonically active interneurons) in the human putamen and macaque caudate nucleus. Counterstaining with DAPI (blue) shows nuclei of all cells. White arrowheads point to double-labeled interneurons. CALB: Calbindin-D-28K, CALR: Calretinin, Cd: caudate nucleus, ChAT: Choline Acetyltransferase, nNOS: Neuronal nitric oxide synthase, PARV: Parvalbumin, Put: putamen, SOM: Somatostatin. Scale bar = 25 μm.

In addition, it is well known that the striatum also contains a small population of medium and/or small-sized GABAergic interneurons, which play an important role in the modulation and control of the MSN, that is, the striatum output. We further performed double labeling IF to study the colocalization of different biomarkers for various subtypes of striatum GABAergic interneurons. Thus, we observed that MCT8 and OATP1C1 are colocalized in human and macaque striatum with the calcium-binding protein PARV in fast-spiking GABAergic interneurons (Figure 8A1–B3 and Figure 9A1–B3) [30], with the calcium-binding proteins CALB (Figure 8C1–D3 and Figure 9C1–D3) and CALR (Figure 8E1–F3 and Figure 9E1–F3), with Neuronal nitric oxide synthase (nNOS) for nitrergic interneurons and persistent and low-threshold spiking interneurons (Figure 8G1–H3 and Figure 9G1–H3) [30,31], and with SOM for persistent and low-threshold spiking interneurons (Figure 8I1–J3 and Figure 9I1–J3 respectively) [30]. As shown in Figure 5, in human and macaque striatum, MCT8 and OATP1C1 also are expressed in multipolar neurons with large soma (around 30 μm in diameter), the typical morphology of large aspiny cholinergic interneurons, which are also called tonically active interneurons [30]. We performed double labeling IF for MCT8/OATP1C1 and the Choline Acetyltransferase (ChAT) as a biomarker for cholinergic interneurons. Figure 8K1–L3 and Figure 9K1–L3 show the coexpression of these transporters with ChAT in humans and macaques. Together, MCT8 and OATP1C1 were observed to be expressed in all these types of striatal interneurons.

When performing immunohistochemistry for OATP1C1, we unexpectedly observed a large number of aggregated spherical vesicles with strong OATP1C1 immunoreactivity in all seven human individuals, congregated in the glial feltwork beneath the ependymal lining of the lateral ventricle nearby the caudate nucleus. Those vesicles vary greatly in size and tend to be located close to the ventricles (Figure S2). These vesicles are not observed in any of the macaque tissue sections analyzed. The OATP1C1 expression in these vesicles is observed, without antigen retrieval, using four different anti-OATP1C1 antibodies against different epitopes of OATP1C1 protein, including the antibody donated by Dr. Visser [48] (Figure S2A), and the antibodies from Bioss, Cusabio, and Abcam. The single mouse-derived sc-398883 Santa Cruz antibody only works with antigen retrieval, and this procedure happens to wash the vesicles. We had found these OATP1C1 vesicles in a previously published study in the motor cortex and we had demonstrated that those aggregations were *Corpora amylacea* [21], vesicular outgrowths produced in astrocytes that could function like entrapping waste products and that can be released from the brain to the cerebrospinal fluid and the lymphatic system [49,50]. To test this in the basal ganglia, we performed double labeling experiments for OATP1C1 and for Immunoglobulin M (IgM) antibodies that recognize the neoepitopes of IgM specifically contained in *Corpora amylacea* [51]. Our results demonstrate that OATP1C1 coexists with IgM in *Corpora amylacea*. The experiments were performed using specific secondary antibodies against the C region of the γ1 chain of rabbit IgG to label rabbit anti-OATP1C1 antibodies, to exclude the possibility of false positives in *Corpora amylacea* immunostaining due to contamination of IgM in commercial antibodies [51].

### 2.4. MCT8 and OATP1C1 Are Expressed in Projection Neurons of Output Nuclei of the Human and Macaque Basal Ganglia

**Figure 10:**
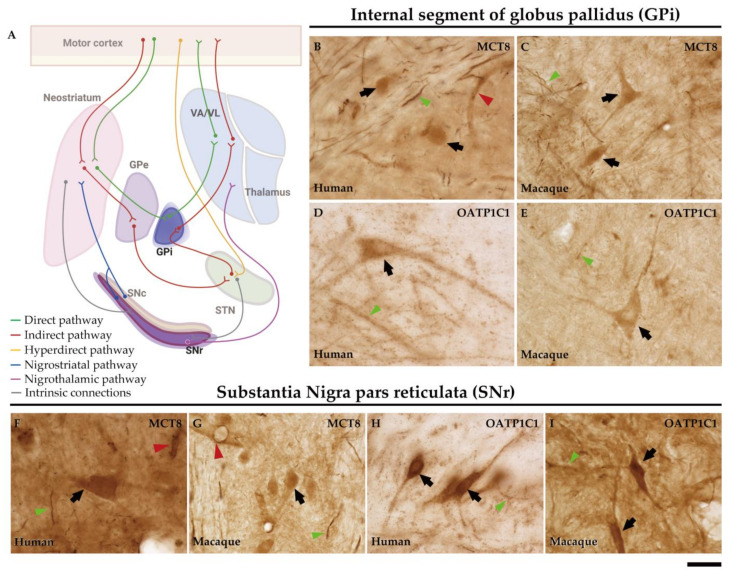
Expression of MCT8 and OATP1C1 in neurons of the output nuclei of the basal ganglia in humans and macaques. (**A**) The diagram summarizes the basal ganglia motor circuits. GPi and SNr serve as output nuclei of the basal ganglia. The axons from GPi innervate the VA/VL of the thalamus. Representative brightfield photomicrographs show immunostaining for MCT8 at the human (**B**) and macaque (**C**) GPi, and at the human (**F**) and macaque (**G**) SNr. OATP1C1 immunostaining is also shown at the human (**D**) and macaque (**E**) GPi and at the human (**H**) and macaque (**I**) SNr. Black arrows point to neurons with immunopositive signals. Note that MCT8, but not OATP1C1, is highly expressed in capillaries (red arrowheads). Both MCT8 and OATP1C1 staining can be observed in fibers (green arrowheads). GPe: external segment of globus pallidus, GPi: internal segment of globus pallidus, SNc: substantia nigra pars compacta, SNr: substantia nigra pars reticulata, STN: subthalamic nucleus, VA/VL: ventral anterior and ventral lateral nuclei of the thalamus. ((**A**) created with BioRender.com). Scale bar = 33 μm (**B**–**E**) and 38 μm (**F**–**I**).

**Figure 11:**
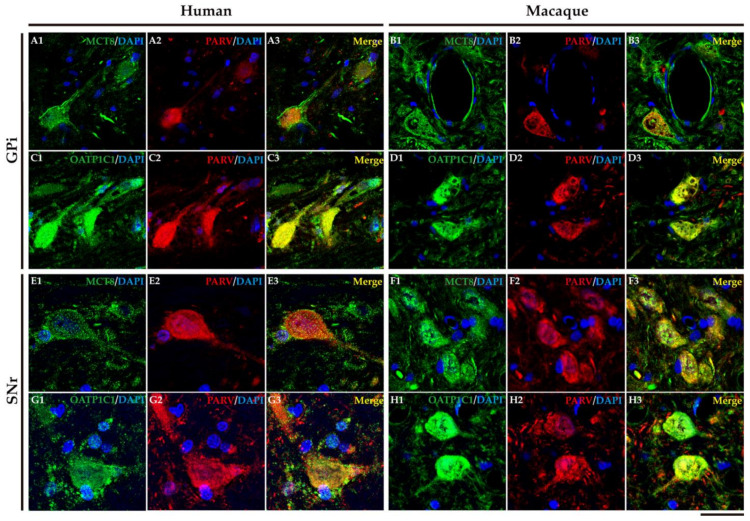
Expression of MCT8 and OATP1C1 in GPi and SNr neurons. Representative confocal microscope compositions of human and macaque sections double stained for MCT8 (green) or OATP1C1 (green) and for the neuron marker PARV, which is specific for GABAergic neurons in the GPi and SNr. Merged images show the colocalization of both markers. MCT8 and PARV are coexpressed in the human (**A1**–**A3**) and macaque (**B1**–**B3**) GPi and in the human (**E1**–**E3**) and macaque (**F1**–**F3**) SNr. OATP1C1 and PARV are coexpressed in the human (**C1**–**C3**) and macaque (**D1**–**D3**) GPi and in the human (**G1**–**G3**) and macaque (**H1**–**H3**) SNr. Counterstaining with DAPI (blue) shows nuclei of all cells. GPi: internal segment of globus pallidus, PARV: Parvalbumin, SNr: substantia nigra pars reticulata. Scale bar = 50 μm (**A1**–**D3**), 25 μm (**E1**–**H3**).

In humans and macaques, the internal segment of globus pallidus (GPi) and substantia nigra pars reticulata (SNr) are the output nuclei of the basal ganglia (Figure 10A). Figure 10B shows that MCT8 immunoreactivity was observed in spherical neurons in human GPi, while Figure 10C, in macaque, shows triangular or fusiform MCT8 immunopositive somas with low branching dendrites, a feature that fits with the typical morphology of the neurons of this nucleus. In addition, many MCT8 immunopositive fibers can be observed in this region in both humans and macaques. OATP1C1 immunoreactive cells show the typical morphology features of the GPi neurons in both humans and monkeys (Figure 10D,E). The double staining IF experiments with PARV showed coexpression with MCT8 and OATP1C1 in those cells (Figure 11A1–D3). The expression of MCT8 and OATP1C1 was also observed in human and macaque SNr (Figure 10F–I). As described previously, the neurons in GPi and SNr are mostly GABAergic and can be labeled by PARV [33,34]. The coexpression of MCT8/OATP1C1 with PARV was confirmed by double labeling IF (Figure 11E1–H3).

### 2.5. MCT8 and OAdTP1C1 Are Expressed in Projection Neurons of the Human and Macaque Intrinsic Nuclei

**Figure 12:**
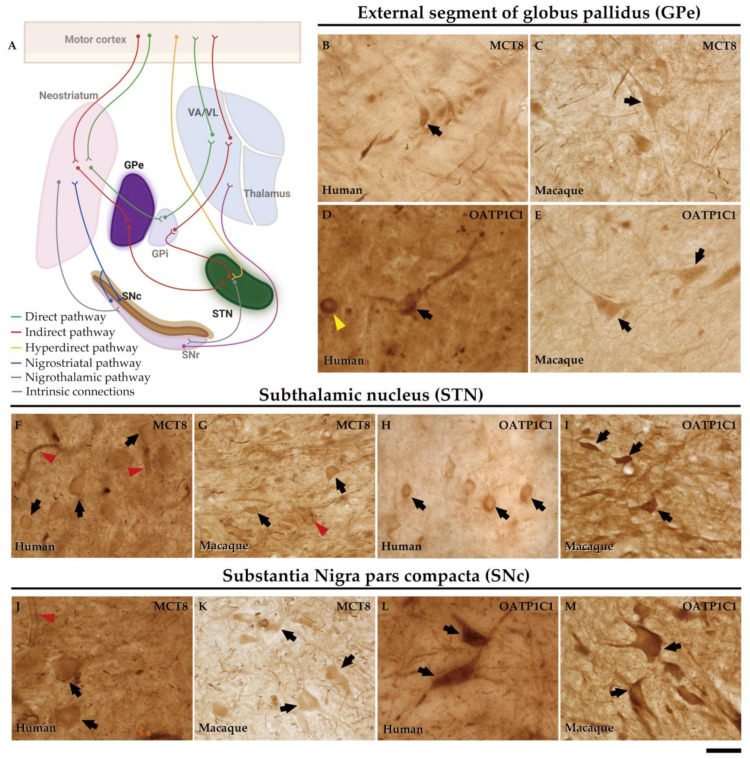
Expression of MCT8 and OATP1C1 in neurons of the intrinsic nuclei of the human and macaque basal ganglia. (**A**) In the motor pathway, GPe, SNc, and STN serve as the intrinsic nuclei of the basal ganglia. GPe is primarily, but not exclusively, used as a relay station between the striatum and the STN. Besides the classical GPe projections, STN also receives projections from the cortex (hyperdirect pathway) and other sources. The projections from SNc innervate the striatum and form the nigrostriatal pathway. Representative brightfield photomicrographs show immunostaining for MCT8 in the GPe of humans (**B**) and macaques (**C**), STN of humans (**F**) and macaques (**G**), and SNc of humans (**J**) and macaques (**K**). OATP1C1 immunostaining is also shown in the GPe of humans (**D**) and macaques (**E**), STN of humans (**H**) and macaques (**I**), and SNc of humans (**L**) and macaques (**M**). Black arrows point to neurons with immunopositive signals. Note that the MCT8 signal, but not the OATP1C1 signal, is highly expressed in capillaries (red arrowheads). Both MCT8 and OATP1C1 staining can be observed in fibers. In addition, an OATP1C1-immunopositive *Corpora amylacea* (yellow arrowhead) can be observed in panel (**D**). GPe: external segment of globus pallidus, GPi: internal segment of globus pallidus, SNc: substantia nigra pars compacta, SNr: substantia nigra pars reticulata, STN: subthalamic nucleus, VA/VL: ventral anterior and ventral lateral nuclei of the thalamus. ((**A**) created with BioRender.com). Scale bar = 33 μm (**B**–**E**) and 38 μm (**F**–**M**).

**Figure 13:**
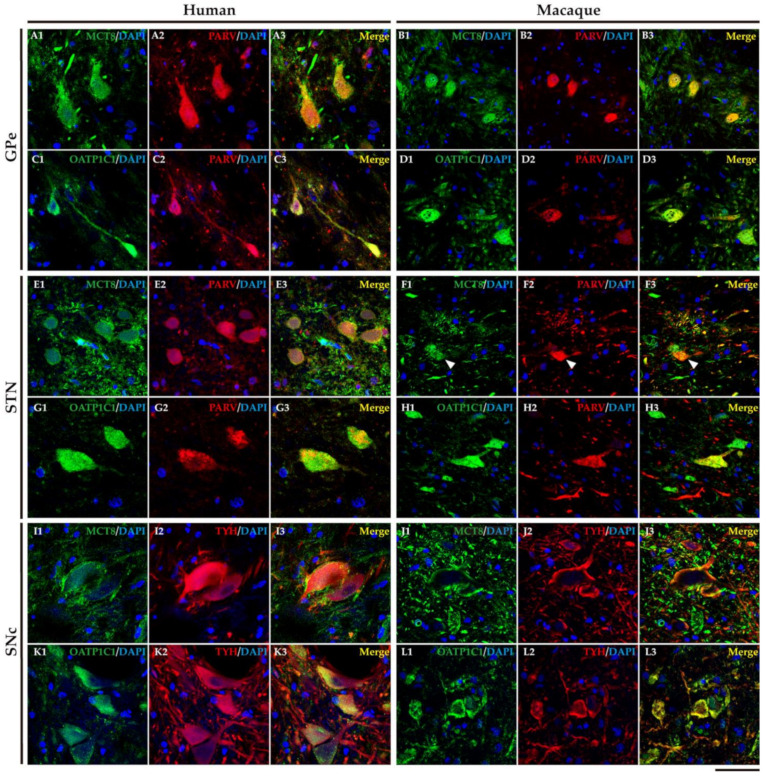
Expression of MCT8 and OATP1C1 in different subpopulations of neurons in intrinsic nuclei of the human and macaque basal ganglia. Representative confocal microscope compositions from double-stained sections for MCT8 (green) or OATP1C1 (green) and the neuron markers PARV (red), and TYH (red). Merged images show the colocalization of both signals. MCT8 and PARV are coexpressed in the GPe in humans (**A1**–**A3**) and macaques (**B1**–**B3**), and in the STN in humans (**E1**–**E3**) and macaques (**F1**–**F3**). MCT8 and TYH are coexpressed in SNc in humans (**I1**–**I3**) and macaques (**J1**–**J3**). OATP1C1 and PARV are coexpressed in the GPe in humans (**C1**–**C3**) and macaques (**D1**–**D3**) and in the STN in humans (**G1**–**G3**) and macaques (**H1**–**H3**). OATP1C1 and TYH are coexpressed in the SNc in humans (**K1**–**K3**) and macaques (**L1**–**L3**). Counterstaining with DAPI (blue) shows nuclei of all cells. GPe: external globus pallidus, PARV: Parvalbumin, SNc: substantia nigra pars compacta, STN: subthalamic nucleus, TYH: Tyrosine hydroxylase. Scale bar = 50 μm.

The external segment of globus pallidus (GPe), subthalamic nucleus (STN), and substantia nigra pars compacta (SNc) of humans and monkeys are the intrinsic nuclei of the basal ganglia (Figure 12A). In the human and macaque GPe, immunoreactivity for both MCT8 and OATP1C1 is present in the soma and dendrites of the typical GPe triangular neurons (Figure 12B–E). Notably, immunopositive signals for MCT8 can be seen in fibers (Figure 12B,C). Double labeling IF experiments confirmed that both TH transporters colocalize with PARV in cells (Figure 13A1–D3) in the human and macaque GPe. (Figure 13A1–B3). Figure 12F–I shows the immunohistochemical signal for MCT8 and OATP1C1 in the human and macaque STN, located in the soma and some dendrites of small neurons. Previous findings indicate that PARV is a biomarker only for projection neurons in the STN [35]. Our double labeling IF experiments show that both TH transporters are coexpressed with PARV in a population of neurons (Figure 13E1–H3), confirming that they are projection neurons. Abundant fibers immunoreactive for MCT8 can be found in this region. In the human and macaque SNc, the expression of MCT8 and OATP1C1 was observed in the soma and dendrites of multipolar and fusiform neurons and fibers (Figure 12J–M). Figure 13I1–L3 shows the results of double staining IF for MCT8 or OATP1C1 with tyrosine hydroxylase (TYH) in the human and macaque SNc, which confirms the presence of both transporters in the dopaminergic neurons. 

### 2.6. MCT8 and OATP1C1 Are Expressed in Thalamocortical Neurons of the Human and Macaque Motor Thalamus

**Figure 14:**
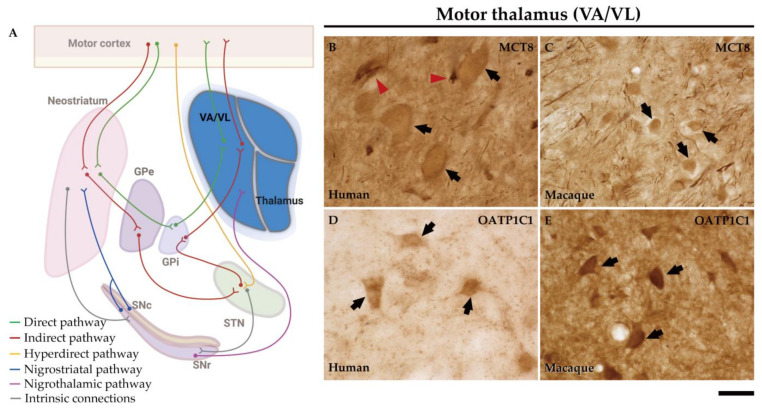
Expression of MCT8 and OATP1C1 in neurons of the motor thalamus. (**A**) The diagram shows that the pathway to the cortex leaves from the GPi to the motor cortex with a relay in the VA/VL of the motor thalamus. Representative brightfield photomicrographs show immunostaining for MCT8 and OATP1C1 in the human (**B**,**D**) and macaque (**C**,**E**) VA/VL. Black arrows point to neurons with immunopositive signals. Note that the MCT8 signal is highly expressed in capillaries (red arrowheads) and fibers, while OATP1C1 can be observed in fibers but rarely in capillaries. GPe: external segment of globus pallidus, GPi: internal segment of globus pallidus, SNc: substantia nigra pars compacta, SNr: substantia nigra pars reticulata, STN: subthalamic nucleus, VA/VL: ventral anterior and ventral lateral nuclei of the thalamus. ((**A**) created with BioRender.com). Scale bar = 33 μm (**B**–**E**).

The ventral anterior and ventral lateral nuclei of the thalamus (VA/VL) of humans and macaques are the motor relay nuclei of the direct and indirect pathways of the basal ganglia to the motor cortex (Figure 14A).

In the human motor thalamus, MCT8 immunocytochemical staining is mainly present in the cell membrane of round medium-sized cells (Figure 14B). In the macaque motor thalamus, MCT8 is expressed at the cell membrane and cytoplasm of neurons (Figure 14C). In both human and macaque motor thalamus, MCT8 immunoreactive fibers are very abundant. The OATP1C1-immunopositive signal is also found at the membrane and cytoplasm of neurons of the human and macaque VA/VL thalamus (Figure 14D,E).

**Figure 15:**
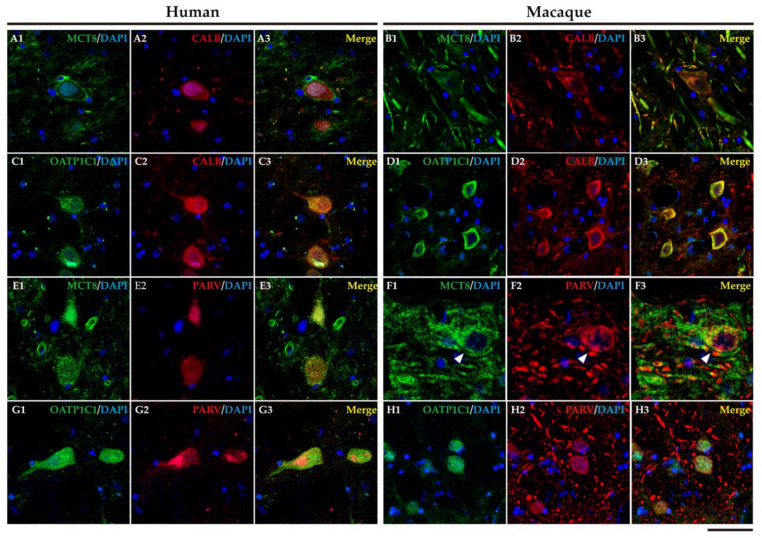
Expression of MCT8 and OATP1C1 in *matrix* cells and *core* cells of the human and macaque motor thalamus. Representative confocal microscope compositions of brain sections double stained for MCT8 (green) or OATP1C1 (green) and the matrix cells marker, CALB (red), or core cells marker, PARV (red). Merged images show the colocalization of both signals. MCT8 is coexpressed with CALB in the human (**A1**–**A3**) and macaque (**B1**–**B3**) VA/VL, and with PARV in the human (**E1**–**E3**) and macaque (**F1**–**F3)** VA/VL (white arrowheads). OATP1C1 is coexpressed with CALB in the human (**C1**–**C3**) and macaque (**D1**–**D3**) VA/VL, and with PARV in the human (**G1**–**G3**) and macaque (**H1**–**H3**) VA/VL. Counterstaining with DAPI (blue) shows nuclei of all cells. CALB: Calbindin-D-28K, PARV: Parvalbumin. Scale bar = 50 μm (**A1**–**E3** and **G1**–**H3**), 25 μm (**F1**–**F3**).

Early studies have shown that CALB- and PARV-expressing neurons (*matrix* cells and *core* cells, respectively) within each thalamic nucleus project to either supragranular or granular layers of the cerebral cortex [37,38,52,53]. We therefore performed the double labeling IF experiment with MCT8 and/or OATP1C1 with those calcium-binding proteins and the results evidenced that both TH transporters colocalize with CALB (Figure 15A1–D3) or PARV (Figure 15E1–H3) being then present in both types of thalamocortical projection neuron (Figure 15). We also found that MCT8 colocalizes with CALB or PARV in thick fibers of the macaque motor thalamus (Figure 15).

### 2.7. MCT8 and OATP1C1 Are Expressed in the Cholinergic Neurons of the Human and Macaque Nucleus Basalis of Meynert (Ventral Pallidum)

**Figure 16:**
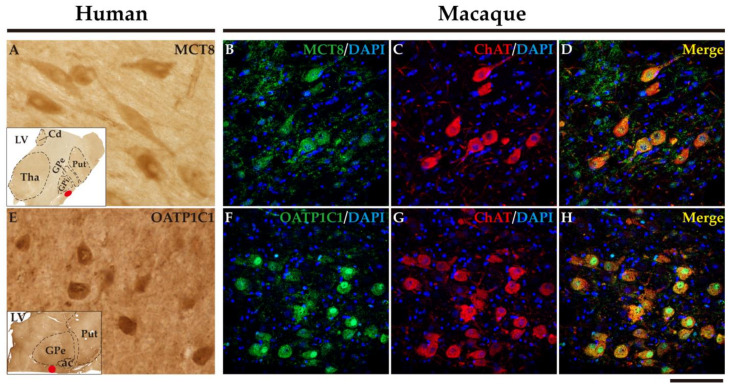
Expression of MCT8 and OATP1C1 in the nucleus basalis of Meynert. Representative brightfield photomicrographs show immunostaining for MCT8 (**A**) and OATP1C1 (**E**) in the human nucleus basalis of Meynert with insets that show the exact location (red points) of the images shown. Representative confocal microscope compositions from double-stained sections for MCT8 (green) or OATP1C1 (green) and the cholinergic neuron marker ChAT (red) in the macaque nucleus basalis of Meynert. Merged images show the colocalization of both signals. (**B**–**D**) Coexpression of MCT8 and ChAT in the macaque nucleus basalis of Meynert. (**F**–**H**) Coexpression of OATP1C1 and ChAT in the macaque nucleus basalis of Meynert. Note that most neurons coexpress both signals. Counterstaining with DAPI (blue) shows nuclei of all cells. ac: anterior commissure, Cd: caudate nucleus, ChAT: Choline acetyltransferase, GPe: external segment of globus pallidus, GPi: internal segment of globus pallidus, LV: lateral ventricle, Put: putamen. Scale bar = 50 μm (**A**,**E**) and 110 μm (**B**–**D**,**F**–**H**).

The nucleus basalis of Meynert is an important source of acetylcholine for the basal ganglia and the cerebral cortex. This nucleus is a relatively diffuse collection of large cholinergic neurons in the human basal forebrain [54]. Figure 16 shows that MCT8 and OATP1C1 immunohistochemical signal is positive in the clusters of large-sized neurons located underneath the inferior limit of the globus pallidus, and between the external and internal segments of that nucleus, where the nucleus basalis of Meynert is found. Figure 16 also shows the results of double labeling IF experiments confirming the coexpression of MCT8 (Figure 16B–D) or OATP1C1 (Figure 16F–H) with ChAT in the macaque brain.

## 3. Discussion

In this study, we have focused on identifying the nature of the neuronal elements of the basal ganglia and motor thalamus that express TH transporters MCT8 and OATP1C1 to understand their importance in the excitation/inhibition balance of the basal ganglia motor circuits. We have performed the study in brain tissues of healthy adult primates, including humans and macaques. Hitherto not reported, our results demonstrate that all types of projection neurons of the various basal ganglia nuclei express both TH transporters. Our research points to the potential consequences of their absence in the basal ganglia circuits as a basis to explain the pathophysiology of MCT8 and OATP1C1 deficiencies, and to provide a foundation of knowledge for future therapeutic applications aimed at cellular targets.

### 3.1. Study Limitations

In the present study, we performed histological analyses of postmortem human brain tissues. Only the human tissue with the best conditions was carefully selected from the brain biobanks based on brain preservation and other aspects related to the optimization of the histological processing. As reflected in Table 1, we ended up mainly with material taken from males. This is a limitation since females are underrepresented in our studies on the distribution of TH transporters. However, the results obtained from the one female of our human sample are the same as those obtained from males. On the other hand, we do have enough females in the monkey sample (4/10), and the results on the cellular distribution of TH transporters are the same for males and females.

The protein expression, antigenicity, and reaction with specific antibodies are susceptible to change whenever the condition of tissue source, cryoprotection, storage conditions and experimental protocols are altered. Regarding the donor status, we selected those brain tissues whose donor ages were either adulthood (29, 32, 55, and 59 years old) or aged (86, 97, and 98 years old) considered normal upon macroscopical and microscopical examination by a pathologist and a neurologist. In murine animal models, expression of MCT10 and OATP1C1 is reduced in the hypothalamus, but not MCT8 [55]. In any case, even if the expression of these transporters had been altered by nonthyroidal illness syndrome in our human tissue, they were still present and both showed a similar immunocytochemical signal in the subpopulations of cells described in the paper, as we have demonstrated consistently, in all the studied brains. Concerning the deleterious effect of the postmortem human brain tissues on antigenicity, we have taken great care to optimize protocols to eliminate nonspecific staining, autofluorescence, and false negatives in order to yield reliable results, as explained in a previous paper [21]. For the same reasons stated above, we only conducted a qualitative study and did not conduct a quantitative study.

The selection of specific antibodies was a critical challenge for this study. The quality and specificity of the primary antibodies used in this work have been verified and discussed in previous articles [21,48,56,57]. The individual variability of the sample and the differences in antibodies might be the reason why most of the human anatomy and histology insights reported in the literature for the MCT8 and OATP1C1 distribution in the basal ganglia are either incomplete or ambiguous. One of the strong points of our study is the careful management of tissues and antibodies to achieve consistent results. According to this study, results among all individuals are indeed similar and reliable, and this is also true for the results obtained for monkeys. When comparing the outcomes between humans and monkeys, the distribution in terms of cell type expression is essentially consistent. Further verification of our findings by other methods, such as in situ hybridization, would be of interest, even though mRNA integrity and preservation in postmortem human tissue are not always guaranteed.

### 3.2. Expression Pattern of TH Transporters in the Human and Nonhuman Primate Basal Ganglia and Thalamus

Our study provides the first immunocytochemical demonstration of the existence of the TH transporters MCT8 and OATP1C1 in the projection neurons of the basal ganglia and motor thalamus, which are those whose axons contribute to the direct and indirect motor pathways of the basal ganglia. Our results are partially supported by GTEx project [41], transcriptomic [42], and northern blot analysis [7] that show mRNA of both MCT8 and OATP1C1 in human basal ganglia, although those studies did not specify which type of neurons were expressing that mRNA. One of the main contributions of our results is the definition of the types of neurons that express the TH transporters MCT8 and OATP1C1 in relation to the activation/inhibition balance of the basal ganglia motor circuits.

In agreement with the previous findings at the protein level by IHC [21,48,56,58,59], we observed the cellular expression pattern of MCT8 in the endothelial cells of vessels, and we demonstrate for the first time the expression of MCT8 in basal ganglia pericytes and the expression of MCT8 and OATP1C1 in monkey choroid plexus. MCT8 had also been found previously in the choroid plexus in the developing human hypothalamus [59].

The functional relevance of MCT8 and OATP1C1 expression at the cellular level in the striatum in primates remains elusive as yet; however, since they are critical for the TH nuclear receptor-mediated signaling [60,61], one might assume that those cells would be affected by some kind of hypothyroidism throughout development and postnatal life. The lack of TH produces severe effects on general brain development such as marked maturational delay in rat caudate nucleus neuronal proliferation [62,63]. That would imply the growing of impoverished MSN neurons and interneurons. On the other hand, it has been reported that the striatum transcriptome profiling of *Slc16a2/Slco1c1* and *Slc16a2/Dio2* double knockout mice overlaps with that of a systemic the hypothyroidism mouse model [64]. However, it is difficult to totally extrapolate this pattern to humans or primate models. Other proteins that have been shown to be regulated by TH specifically in the striatum might also be affected by the deficiency of TH transporters. For instance, the expression of the synaptic plasticity TH target genes, such as Calmodulin Kinase II, RC3/neurogranin, and Rhes (a TH-induced gene that regulates the striatal motor activity and promotes tunneling nanotube transport [65]), is reduced in the hypothyroid rodent models, RC3/neurogranin and Rhes, both being present in the MSN neurons of the striatum [66,67]. Although there are differences between the clinical profiles of hypothyroidism and TH transporters deficiency, it is plausible that all or some of those proteins might also be affected by the deficiency of TH transporters in the MSN, resulting in defects in the neuronal synaptic transmission throughout the basal ganglia motor circuits.

The possibility that MSN were hypotrophic owing to the deficient TH transport across their cytoplasmic membrane due to MCT8 or OATP1C1 deficiency is important since these neurons are the origin of the basal ganglia motor circuits. In addition, as we have demonstrated in this study, the interneurons that modulate the activity of MSN, such as the cholinergic, CALR, PARV, nNOS, and SOM ones, also express MCT8 and OATP1C1 and, therefore, they would also be vulnerable to the lack of TH.

### 3.3. TH Transporters in the Nucleus Basalis of Meynert

This is the first demonstration of MCT8/OATP1C1 transporters in the cholinergic cells of the nucleus basalis of Meynert. The functional consequence of this finding is the sensitivity of those neurons to TH regulation.

TH participate in the development of the cholinergic system [68,69,70] and in the maintenance of the nucleus basalis of Meynert [71]. Cholinergic basal forebrain neurons are sensitive to TH during development [72]. Administration of T3 leads to an earlier and increased expression of cholinergic markers in the caudate–putamen complex [69] and the nucleus basalis of Meynert [72]. In addition, several data indicate a close link between TH and neurotrophins in the basal forebrain [69,70]. Immunostaining for nerve growth factor receptor, which is expressed in ChAT-positive neurons in the basal forebrain, is also regulated by TH content [69]. Furthermore, some studies show a reduction of ChAT mRNA in the basal forebrain of adult onset hypothyroid rats indicating a role for TH in the maintenance of cholinergic neurons during adulthood [73]. A reduction of TH levels was also reported to induce nucleus basalis of Meynert cell death [71]. Trophic actions of TH on the cholinergic system have been described through the stimulation of the acetylcholine metabolism, the increase of acetylcholinesterase activity, and acetylcholine uptake [74]. Moreover, TH were reported to regulate muscarinic receptor expression, ligand affinity [75], and acetylcholinesterase expression [76]. In the CNS, cholinergic signaling is critical to many cognitive processes [77,78,79]. TH may directly regulate cholinergic signaling by controlling the expression of ChAT, the gene responsible for the synthesis of acetylcholine, by TRs [80]. Several in vitro and in vivo studies have correlated TH signaling with ChAT expression, particularly during development [80,81,82,83]. In the case of TH transporter deficiency, it could be assumed that the cholinergic neurons of the nucleus basalis of Meynert would be affected in their development, function, and signaling, resulting in disturbances in many systems that are critical for learning, memory, and synaptic plasticity.

### 3.4. Model of TH Transport in Human and Nonhuman Primate Neostriatum Microcircuitry

**Figure 17:**
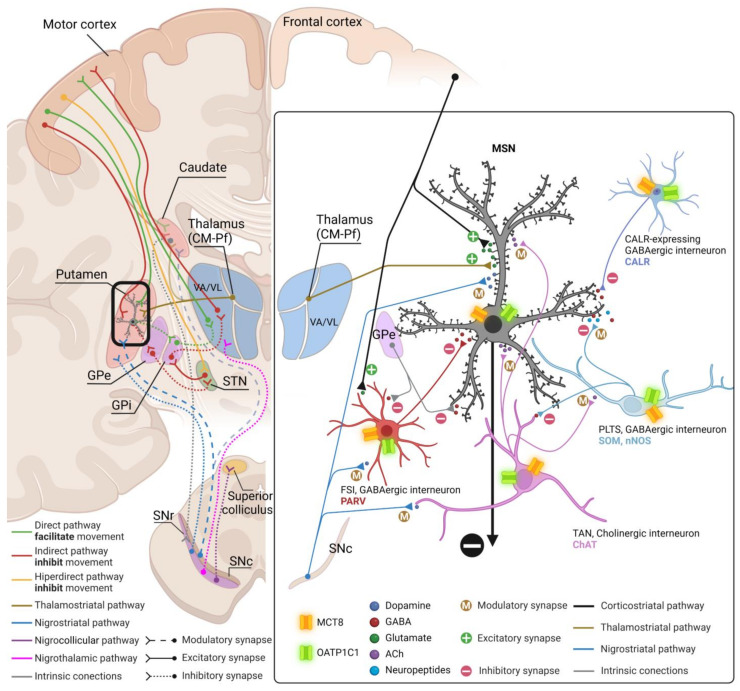
Model of TH transporters in human and primate neostriatum microcircuitry based on our findings. The (**left panel**) summarizes the main circuits of the basal ganglia, and the (**right panel**) depicts the local microcircuit that occurs in the neostriatum (black frame on the left panel) with the expression of TH transporters in its components and their specific neurotransmitters. (**Left**): the neocortex projects directly to the striatum (caudate and putamen) cells via the corticostriatal pathway (glutamatergic). MSN (most of the striatum cells) and some types of interneurons receive this excitatory cortical input. MSN are GABAergic projection neurons. The CM-Pf nuclei of the thalamus provide other major excitatory glutamatergic input to MSN via the thalamostriatal pathway. Additionally, the striatum receives the projections of dopaminergic cells from the SNc via the nigrostriatal pathway in MSN and in other types of interneurons. Interneurons are either cholinergic or GABAergic and participate in the local microcircuitry that modulates MSN activity. The MSN integrate all this information and produce two different signaling pathways: the direct pathway, in which MSN send inhibitory projections to the GPi, which in turn disinhibit the thalamus activating the thalamocortical system and therefore movement, and the indirect pathway, where MSN first inhibit the GPe, followed by disinhibition of the STN, which then excites GPi inhibiting the thalamus and thence movement. Finally, the VA/VL nuclear complex of the Thalamus is the primary target of the GPi, relaying the modulatory effects of the basal ganglia to the upper motor neurons in the cortex. The SNr sends axons to the thalamus and to the superior colliculus. (**Right**): The right-hand diagram shows the distribution of the transporters MCT8 (orange) and OATP1C1 (green) in all types of cells of the striatal local microcircuitry in the human and macaque. TH are involved in modulating the output of MSN and the activity of all the neurons related in the corticostriatal, thalamostriatal, and nigrostriatal pathways controlling movement. Ach: acetylcholine, CM-Pf: centromedian–parafascicular nuclei of the thalamus, FSI: fast-spiking GABAergic interneurons, GABA: γ-Aminobutyric acid, GPe: external segment of globus pallidus, GPi: internal segment of globus pallidus, MSN: medium-sized spiny neurons, PLTS: persistent and low-threshold spiking interneurons, SNc: substantia nigra pars compacta, SNr: substantia nigra pars reticulata, STN: subthalamic nucleus, TAN: tonically active interneurons, VA/VL: ventral anterior and ventral lateral nuclei of the thalamus (created with biorender.com).

Based on our novel findings regarding the cellular location of MCT8 and OATP1C1 in the human and monkey basal ganglia and motor thalamus, Figure 17 depicts our proposed model of TH transport within the motor pathways and striatum microcircuitry.

The striatal MSN are the first station of the basal ganglia system of circuits being the recipients of the efferent projections from the cortex and the thalamus and being modulated by local interneurons and by other afferents from the substantia nigra, amygdaloid complex, raphe nuclei, and parabrachial pontine reticular formation. It is well known that the MSN recorded in vivo fire few action potentials spontaneously [84,85,86], and that their activity is regulated by extrinsic inputs as well as by the strong feedforward from interneurons. The dysfunction of the striatal microcircuitry because of a lack of MCT8 or OATP1C1 transporters may be another factor that contributes to the pathophysiology of the movement disorders that appear in TH transporter deficiency syndromes.

In this paper, motor functions related to the basal ganglia are discussed in relation to the expression of the TH transporters MCT8 and OATP1C1 and to the possible effects of their deficiency in the cortical–basal ganglia–thalamus–cortical motor loop (Figure 17). In a previous article, we also proposed a pathogenesis model for OATP1C1 and MCT8 deficiency syndromes related to the anatomopathological effects in the neuronal subpopulations of the motor cortex (pyramidal neurons and interneurons), which also express these transporters [21]. However, other types of sensorimotor, associative, and limbic information flow in parallel streams through the basal ganglia–thalamocortical loop [87]. How nonmotor basal ganglia channels are affected in AHDS- or OATP1C-deficient patients, and whether their alterations are correlational in the cognitive and behavioral symptoms, remains a potential area for further investigation.

## 4. Materials and Methods

### 4.1. Human Samples and Tissue Preparation

The present results were obtained by analyzing postmortem samples obtained from seven healthy individuals, whose ages ranged from 29 to 98 years old and who did not exhibit any neurological or psychiatric disorder, as detailed in Table 1. The material was generously provided by Dr. Ricardo Insausti from the Human Neuroanatomy Laboratory at the University of Castilla-La Mancha in Albacete, Spain, and Dr. Lucía Prensa from the Department of Anatomy, Histology, and Neuroscience at the Autónoma de Madrid University in Madrid, Spain.

All human brain tissue studied for this paper was prepared with the same special protocol for neuroscience studies. The data on the length of admission in the hospital or intensive care unit were not reflected in the death certificate at the time, but all the brains arrived at the necropsy laboratory between 2 and 24 h postmortem. The necropsy was performed by a pathologist and a neurologist, and careful macroscopical and microscopical examination of samples of brain tissue was performed by the pathologist to discard edema, hemorrhages, infections, or metastasis and to ensure that the brain was normal and could be used as a control sample. Tissue from donors of the same list and protocol has been used in other published papers such as Iglesias et al., 2018 [88], Uroz et al., 2004 [89], and Wang et al., 2023 [21]. The tissue preparation has been extensively reported elsewhere [21,29,88,89]. Briefly, the brains were cut into thin blocks that were fixed by immersion in 4% paraformaldehyde for 10 days or 10% buffered formalin for at least 4 weeks. Blocks of brains were immersed in 15% sucrose at 4 °C until they sank before cutting. Samples were cut with a freezing microtome into 50 μm thick coronal sections that were serially collected in a cryoprotective solution and stored at −80 °C.

### 4.2. Monkey Samples and Tissue Preparation

We obtained tissue samples from 10 healthy adult monkeys (Table 2), which had been stored in Dr. Rausell’s tissue bank located in the Department of Anatomy, Histology, and Neuroscience at the Autónoma de Madrid University in Madrid, Spain, and preserved using cryoprotective techniques.

The tissue preparation method used in this study has also been described in detail in our previous studies [21,90]. To summarize, the monkeys were anesthetized using intramuscular ketamine and intravenous Nembutal, after which they were perfused with normal saline and a solution of 4% paraformaldehyde and 1% glutaraldehyde in phosphate buffer through the ascending aorta. The brain was then removed and blocked, and the blocks were postfixed in 4% paraformaldehyde for 4 h, followed by infiltration with 30% sucrose in 0.1 M PB at 4 °C with gentle stirring, freezing in dry ice, and storage at −80 °C. The tissue was cut into 25–30 μm thick coronal sections tangential to the pia mater using a freezing sliding microtome, and alternate series of sections were collected in a sterile cryoprotectant solution and stored in a −80 °C freezer. These series were to be processed later for Nissl staining, IHC, and double/triple labeling with IF for several neuronal and vascular biomarkers.

### 4.3. Histological Analysis

After the above procedures, one series was selected for Nissl staining with thionine to study the cytoarchitectonics and to outline the borders of basal ganglia and thalamic nuclei. This series would serve as a reference for the topographical location of elements labeled by means of IHC and double IF for MCT8/OATP1C1 and several cell/vessel markers in adjacent sections. The different nuclei were identified by anatomical landmarks, such as ventricles, together with the indications of the atlas of the human brain by Mai et al., 2015 [44] associated with details previously described for the thalamus by Iglesias et al., 2018 [88] for humans, and the atlas from Winters et al., 1969 [45] and Emmers et al., 1963 [91] for monkeys.

### 4.4. Immunohistochemistry (IHC)

IHC and double IF procedures and the evaluation of the specificity of antibodies were performed as previously described [21] in free-floating tissue sections.

Briefly, for detection of the MCT8 TH transporter, we used a rabbit polyclonal anti-MCT8 antibody (IHC 1:700–1:2000/IF 1:600, Sigma-Aldrich HPA003353, St. Louis, MO, USA) that had been validated previously in human tissue [56]. For detection of the OATP1C1 TH transporter, we used antibodies from five different suppliers: four rabbit polyclonal, human-derived, anti-OATP1C1 (IHC 1:200–1:500/IF 1:200, hOATP1C1-3516, an antibody donated by Dr. T. Visser [48]; IHC 1:500/IF 1:200, bs-11436R, Bioss; IHC 1:600/IF 1:300, CSB-PA868355LA01HU, Cusabio; IHC 1:500/IF 1:300, ab234729, Abcam, Boston, MA, USA) and a mouse monoclonal, mouse-derived, anti-OATP1C1 (IHC 1:50, sc-398883, Santa Cruz Biotechnology, Santa Cruz, CA, USA) that had been previously used in human and monkey brain tissue [21,48]. The OATP1C1 antibody from Dr Visser is the only one that had been validated in transfected cells with OATP1C1 or with the empty vector [48]. We worked hard to define the specificity of other commercial antibodies, taking the results obtained previously with the antibody donated by Dr Visser as reference; that is, confirming immunopositive signals at the plasma membrane, cytoplasm, cell processes, and choroid plexus and sometimes at the nucleus and nucleolus [48]. In Figure S3, we show the results obtained after the use of all the commercial anti-OATP1C1 antibodies in IHC-stained sections of the cortex that are easier to manage than those of the diencephalon. After numerous experiments with different titrations, and with several antigen retrieval trials, we concluded that Bioss, Cusabio, and Abcam antibodies produce specific results similar to Dr Visser’s, while the Santa Cruz antibody worked for human tissue only after applying techniques of antigen retrieval. Our results are based on the use of all the antibodies.

In some of the experiments, the unmasking of epitopes was performed for 30 min incubating human tissue at 90 °C or macaque tissue at 37 °C in pH 6.0 sodium citrate buffer or pH 8.0 Tris–EDTA buffer. Inactivation of endogenous peroxidases was also performed by incubating the sections in 10% H_2_O_2_ and 10% methanol for 15 min twice. Then, sections were incubated in 5% normal serum of the same species as that in which the secondary antibody was raised [21], 4% bovine serum albumin (BSA), and 0.1 M Lysine in 0.1 M PB as a blocking buffer for 60 min. The primary antibodies were diluted in 0.1 M PB containing 1% normal serum, and 4% BSA and were incubated with the tissue sections for 48 h at 4 °C on a shaking platform. The biotinylated secondary antibodies (Table S1) were diluted in the same solution as the primary antibody and incubated for 1 h at room temperature. We mainly used the avidin–biotin complex method (ABC, Invitrogen, Carlsbad, CA, USA, 32050) for 60 min for signal amplification. Immunoreactivity was developed by 0.5 mg/mL 3,3′-5,5′-diaminobenzidine tetrahydrochloride (DAB, Sigma, D5905) and 0.01% H_2_O_2_ in 0.1 M PB. Finally, sections were mounted on gelatin-coated glass slides, dehydrated in a series of graded dilutions of ethanol to xylene, and coverslipped with DPX.

### 4.5. Double/Triple Immunofluorescence (IF)

Double/triple IF sections were used for the colocalization of MCT8 or OATP1C1 immunoreactivity with different biomarkers or lectins. The antibody against PDGFR-β (IHC 1:200/IF 1:100, AF385, R&D systems, Minneapolis, MN, USA) was used to label all vascular mural cells [92]; the lectin UEA-I was used to label blood vessel endothelial cells [93]; anti-DRD1 (IHC 20 μg/mL/IF 25 μg/mL, MAB8276, R&D systems) labeled D1-MSN in the direct pathway and anti-DRD2 (IHC 20 μg/mL/IF 25 μg/mL, MAB9266, R&D systems) labeled D2-MSN in the indirect pathway [27]; the antibody against the calcium-binding protein CALB (IHC 1:2000/IF 1:1000, #C9848, Sigma-Aldrich) was used to label striatal GABAergic interneurons [29] and *matrix* projection cells in the thalamus [37,38,94]; the antibody against the calcium-binding protein CALR (IHC 1:2000/IF 1:1000, 6B3, Swant, Surry Hills, Australia) labeled GABAergic interneurons in neostriatum [29]; the antibody against ChAT (IHC 1:500/IF 1:250, AB144P, Merck Millipore, Burlington, MA, USA) labeled cholinergic interneurons in neostriatum [29] and cholinergic neurons in nucleus basalis of Meynert [54]; the antibody against PARV (IHC 1:2000/IF 1:1000, #P3088, Sigma-Aldrich) labeled striatal GABAergic interneurons [29], GABAergic neurons in GPe, GPi and SNr [32,33,95], projection neurons in STN, and *core* projection cells in thalamus [37]; the antibody against nNOS (IHC 15 μg/mL/IF 10 μg/mL, AF2416, R&D systems) labeled GABAergic interneurons in neostriatum [31]; the antibody against SOM (IHC 1:500/IF 1:300, sc-55565, Santa Cruz Biotechnology) labeled GABAergic interneurons in neostriatum [30]; and finally the antibody TYH (IHC 1:400/IF 1:200, sc-25269, Santa Cruz Biotechnology) labeled dopaminergic neurons in SNc [95].

Before performing the additional procedures for double IF staining, the tissue was subjected to 48 h of photobleaching pretreatment to bleach endogenous autofluorescence. For unmasking the epitope and blocking unspecific signals, we used the same method as mentioned above for IHC. Then, sections were treated for 72 h at 4 °C with a solution of 0.1 M pH 7.4 PB, 4% BSA, 1% normal serum plus the primary antibodies followed by several cold washes and by 3 h of incubation in the same solution, but this time with fluorescent dye-conjugated (AF488, AF546, AF647 Invitrogen) secondary antibodies (Table S1) against the species of the primary antibody [21]. Finally, the sections were immersed for 5 min in a solution containing 0.01% 4′, 6-diamidino-2-phenylindole (DAPI; Invitrogen, D1306) to label cellular nuclei, and coverslipped with FluorSave (Merck, 345789).

To specifically label *Corpora amylacea*, we performed double labeling experiments using an IgM immunoglobulin that recognizes IgM neoepitopes contained in the vesicles [51] and anti-OATP1C1. To eliminate the possibility of false positives caused by IgM contaminants in commercial OATP1C1 antibodies, which had previously been reported [51], we used secondary antibodies against the γ1 chain C region of the primary rabbit IgG (1:200, OASB01959, Aviva Systems Biology, San Diego, CA, USA) to specifically recognize the primary anti-OATP1C1 antibodies. Sections were incubated for 72 h at 4 °C with OATP1C1 antibodies and human IgM (1:25, I8260, Sigma-Aldrich) and for 3 h with a fluorescein-conjugated secondary antibody (Table S1) at 4 °C. The sections were immediately washed, counterstained with 0.01% DAPI for 5 min, and coverslipped with FluorSave.

### 4.6. Image Acquisition and Processing

Brightfield microscope images of IHC-stained sections were analyzed with Neurolucida software (Version 2022, MicroBrightField Bioscience, Williston, VT, USA). Images were captured with the following objectives: Nikon Plan Apo 2X/0.1; Plan Flour 10X/0.30 DIC L; Plan Flour 20X/0.50 DIC M; Plan Flour 40X/0.75 DIC M; and Plan Apo 60X/1.40 oil DIC H with a digital camera attached to a Nikon microscope (Nikon Eclipse 400, Nikon Instech Co., Ltd., Kawasaki, Japan). Plots of the location of the immunostained cells were generated with 3D Neurolucida at 40X to illustrate the general topographic distribution of the immunostained cells. Immunofluorescent images were captured with the following objectives: ZEISS Plan-APOCHROMAT: 25X/0.8 Imm Korr DIC, 40X/1.3 Oil DIC, and 63X/1.40 Oil DIC using a Zeiss confocal microscope (Zeiss, Spectral Confocal Microscope LSM710, Oberkochen, Germany) and Zen software (version 3.1 pro). Image analysis and treatment, including merging of images, contrast and brightness modification, and maximum intensity projection, were performed using FIJI software (version 2.9.0/1.53t, date: 14 September 2022, U. S. National Institutes of Health, Bethesda, MD, USA). Control or reference images were acquired under the same conditions as the respective set of images.

## Figures and Tables

**Table 1:** Clinical data of human donors.

Sex	Age (y)	Postmortem Interval (h)	Brain Weight (g)	Cause of Death
Male	29	4	1500	Lung tumor
Male	32	3	1420	Hemorrhagic gastroenteritis
Male	54	12	1350	Aortic aneurysm
Male	59	<24	1020	Pneumonia
Male	86	<24	Not available	Not available
Male	97	9	1238	Septic shock
Female	98	6	1168	Not available

**Table 2:** Data of monkey brain tissue.

Species	Age (y)	Sex
*M. fascicularis*	3	Female
*Saimiri sciureus*	3	Female
*M. fascicularis*	5	Male
*M. fascicularis*	5	Male
*M. fascicularis*	5	Female
*M. fascicularis*	5	Male
*M. fascicularis*	5	Male
*M. fascicularis*	5	Female
*M. fascicularis*	5	Male
*M. mulatta*	7	Male

## Data Availability

Not applicable.

## Supplementary Materials

The following supporting information can be downloaded at: https://www.mdpi.com/article/10.3390/ijms24119643/s1.
