# Supplementary material for: Thyroid Hormone Transporters MCT8 and OATP1C1 Are Expressed in Projection Neurons and Interneurons of Basal Ganglia and Motor Thalamus in the Adult Human and Macaque Brains
Source: Int J Mol Sci. 2023 Jun 1;24(11):9643. doi: 10.3390/ijms24119643 (PMC10254002; doi:10.3390/ijms24119643)
Supplement: Supplementary file 1 [file ijms-24-09643-s001.zip › ijms-2380738-supplementary.pdf]

Type of the Paper (Article)

Thyroid Hormone Transporters MCT8 and OATP1C1 Are Expressed in Projection Neurons and Interneurons of Basal Ganglia and Motor Thalamus in the Adult Human and Macaque Brain

Ting Wang [1, 3, †], Yu Wang [1, 3, †], Ana Montero-Pedrazuela [2], Lucía Prensa [1], Ana Guadaño-Ferraz [2,*], Estrella Rausell [1,*]

[1] School of Medicine, Dept Anatomy Histology & Neuroscience, Autónoma de Madrid University (UAM), 28029 Madrid, Spain.
[2] Instituto de Investigaciones Biomédicas Alberto Sols, Consejo Superior de Investigaciones Científicas (CSIC)-Autónoma de Madrid University (UAM), 28029 Madrid, Spain.
[3] PhD Program in Neuroscience, Autónoma de Madrid University (UAM)-Cajal Institute, 28029 Madrid, Spain.
† These authors contributed equally to this work.
* Correspondence: ER: estrella.rausell@uam.es; AG-F: ana.guadano.ferraz@csic.es, aguadano@iib.uam.es.

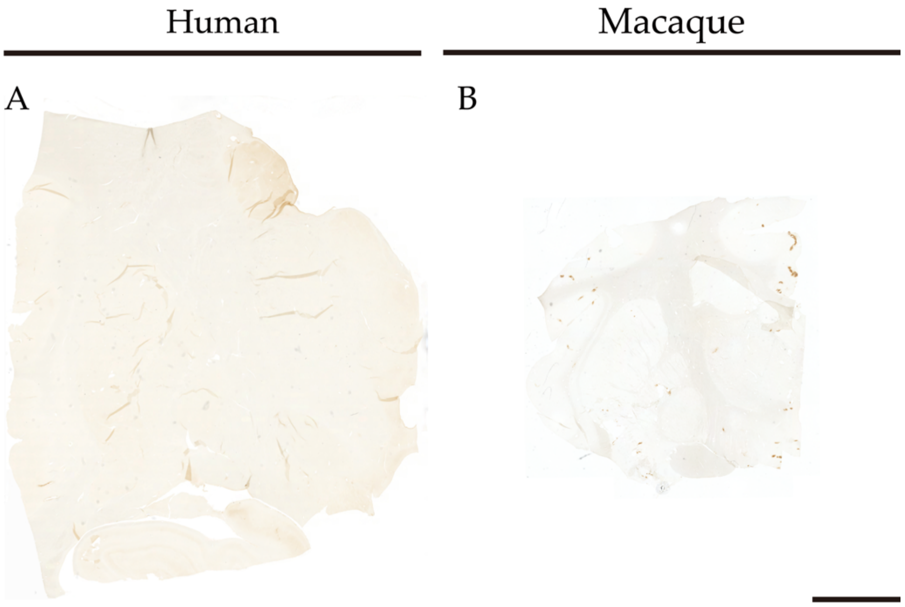

Figure S1. Negative control sections for the immunostaining protocol in human and macaque brain tissue. Representative brightfield photomicrographs show the results of the immunostaining procedure without the primary antibody in human and macaque brain tissue in sections that had been processed at the same time that those shown in the main text. (**A**) The caudate nucleus has a slightly nonspecific background, and (**B**) nonspecific DAB deposits can be seen at the tissue's edge or at the break caused by over-time DAB developing, however, no neural or capillary immunoreactivity is detected in human (**A**) or macaque (**B**) brain tissue sections without using primary antibodies. Scale bar = 8000 μm (A) 6000 μm (B).

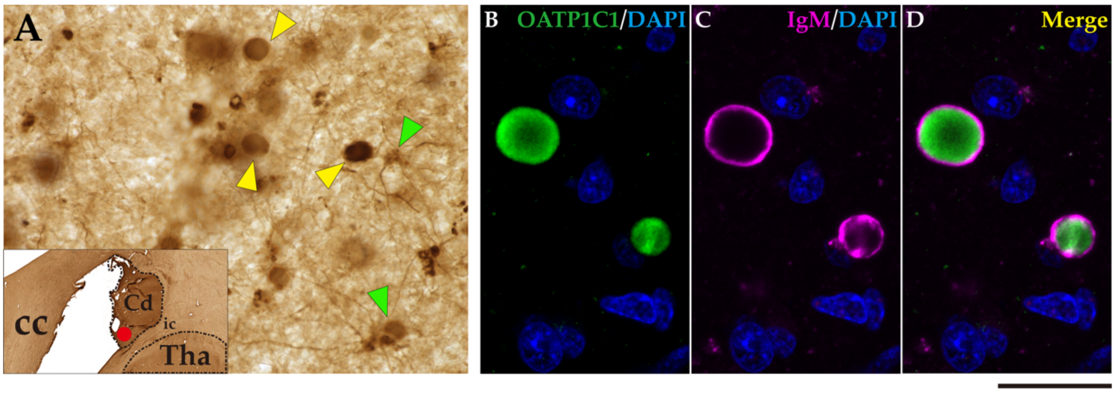

Figure S2. Expression of OATP1C1 in Corpora amylacea at the human neostriatum. Representative brightfield photomicrographs showing immunostaining for OATP1C1 in the human caudate nucleus. (**A**) Expression OATP1C1 in *Corpora amylacea* (yellow arrowheads) on the border of the caudate nucleus and ventricle. The high magnification image is extracted from the location at the red point in the lower magnification inset. Note some OATP1C1 immunopositive astrocyte-like cells (green arrowhead). (**B**-**D**) Confocal microscope images of double labeling for OATP1C1 (green, **B**), and the *Corpora amylacea* marker IgM (purple, **C**). The merged image (**D**) shows the colocalization of the OATP1C1 and IgM. Counter-staining with DAPI (blue) shows nuclei of all cells. cc: corpus callosum, Cd: caudate nucleus, ic: internal capsule, Tha: thalamus. Scale bar = 50 µm (A) and 20 µm (B-D).

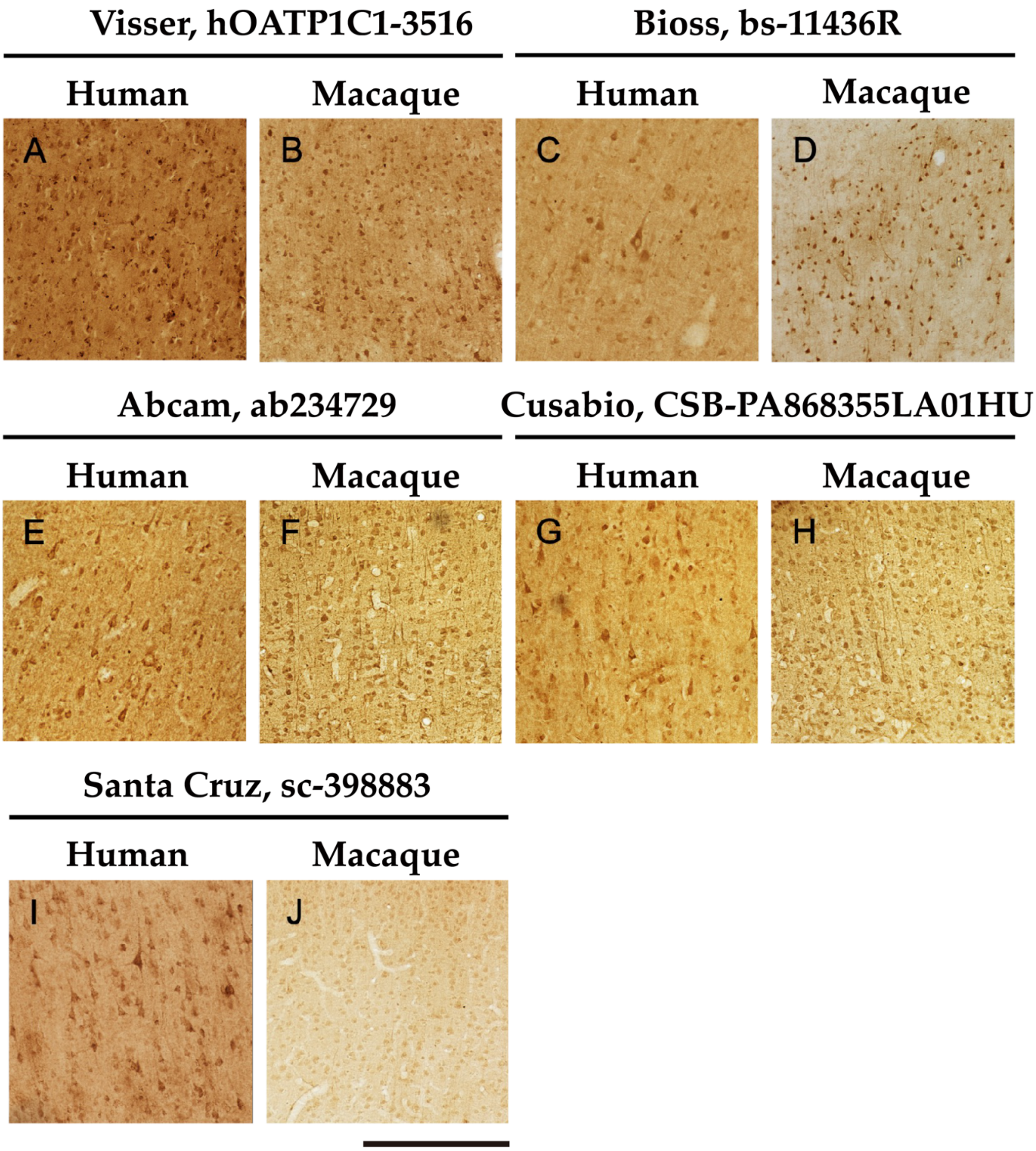

Figura S3 Validation of anti-OATP1C1 antibodies. **A**-**J** show immunopositive signals with anti-OATP1C1 antibodies from Visser (hOATP1C1-3516) (**A**, **B**), Bioss (bs-11436R) (**C**, **D**), Abcam (ab234729) (**E**, **F**), Cusabio (CSB-PA868355LA01HU) (**G**, **H**), and Santa Cruz (sc-398883) (**I**, **J**) in layers III-V of human and monkey cortex. Similar results were obtained using these 5 antibodies, except that the Santa Cruz antibody very weakly labels monkey cortex tissues (**J**). Additionally, IHC with Santa Cruz antibody shown in **I** and **J** required antigen retrieval. All antibodies can label multiple types of neurons, including large numbers of pyramidal neurons. Compared with the other antibodies, the antibody from Dr. Visser shows more background noise in human cortex (**A**). The bar represents 200 μm (A-J).

Table S1: List of secondary antibodies used in this study.

Antibody	Catalog #	Supplier	IHC Dilution	IF Dilution
Goat anti-Rabbit Biotinylated	BA-1000	Vector Laboratories	1:200	-
Horse anti-Mouse Biotinylated	BA-2000	Vector Laboratories	1:200	-
Rabbit anti-Rat Biotinylated	BA-4000	Vector Laboratories	1:200	-
Goat anti-Rabbit Ig gamma-1 chain C region Biotinylated	OASB01959	Aviva Systems Biology	1:200	-
Goat-anti-Mouse IgG (AF647)	A21236	Invitrogen	-	1:500
Goat-anti-Mouse IgG (AF546)	A11030	Invitrogen	-	1:500
Goat-anti-Rabbit IgG (AF488)	A11034	Invitrogen	-	1:500
Donkey-anti-Rabbit IgG (AF488)	A21206	Invitrogen	-	1:500
Donkey-anti-Rabbit IgG (AF647)	A31573	Invitrogen	-	1:500
Donkey-anti-Goat IgG (AF488)	A11055	Invitrogen		1:500
Donkey-anti-Goat IgG (AF546)	A11056	Invitrogen	-	1:500
Donkey-anti-Mouse IgG (AF647)	A31571	Invitrogen	-	1:500
Donkey-anti-Mouse IgG (AF546)	A10036	Invitrogen	-	1:500
Goat anti-Human IgM (AF647)	A21249	Invitrogen	-	1:200

IHC, immunohistochemistry; **IF**, immunofluorescence.
